# Transient regulatory T cell manipulation is limited by anti-antibody responses in HIV-1 envelope immunized rhesus macaques

**DOI:** 10.1016/j.isci.2025.113191

**Published:** 2025-07-23

**Authors:** Shuqin Gu, Kan Luo, Tarra A. Von Holle, Thaddeus C. Gurley, Hilary Bouton-Verville, Laura L. Sutherland, Robert Parks, Xiaoying Shen, Rachel L. Spreng, Georgia D. Tomaras, David C. Montefiori, Hua-Xin Liao, Barton F. Haynes, M. Anthony Moody

**Affiliations:** 1 Duke Human Vaccine Institute, Duke University School of Medicine, Durham, NC 27710, USA; 2 Department of Surgery, Duke University School of Medicine, Durham, NC 27710, USA; 3 Department of Integrative Immunobiology, Duke University School of Medicine, Durham, NC 27710, USA; 4 Department of Medicine, Duke University School of Medicine, Durham, NC 27710, USA; 5 Department of Pediatrics, Duke University School of Medicine, Durham, NC 27710, USA

**Keywords:** Biological sciences, Immunology, Virology

## Abstract

CD25^+^ FoxP3^+^ CD4^+^ regulatory T (Treg) cells promote immune tolerance. We studied germinal center responses and HIV-1 antibody development in rhesus macaques (RMs) immunized with sequential CH505 gp120 envelopes (Envs), with or without anti-CD25 monoclonal antibody (mAb). Plasma Env antibody levels and CD4 binding sited-directed responses were similar across groups. Treg and CXCR5-expressing follicular Treg cell frequency dropped more than two times after the first anti-CD25 infusion but not later ones. Transient Treg disruption was associated with a reduced proportion of vaccine-elicited B cell clonal lineages in lymphoid tissue, but did not result in neutralization breadth. Anti-CD25-treated RMs developed anti-drug antibodies, correlating with reduced plasma mAb levels after subsequent infusions. Germinal center responses were modified by Treg perturbation intended to induce HIV-1 bnAbs, but this effect was curtailed by anti-antibody responses. This may have implications for vaccination in persons receiving immune modulating drugs for transplants or other medical conditions.

## Highlights

• Anti-CD25 treatment briefly perturbs Treg cell frequency in HIV Env-immunized rhesus macaques
• Treg disruption alters germinal center responses but does not enhance antibody breadth
• Anti-CD25-induced Treg perturbation is constrained by anti-drug immune responses

## Graphical abstract

**Figure:**
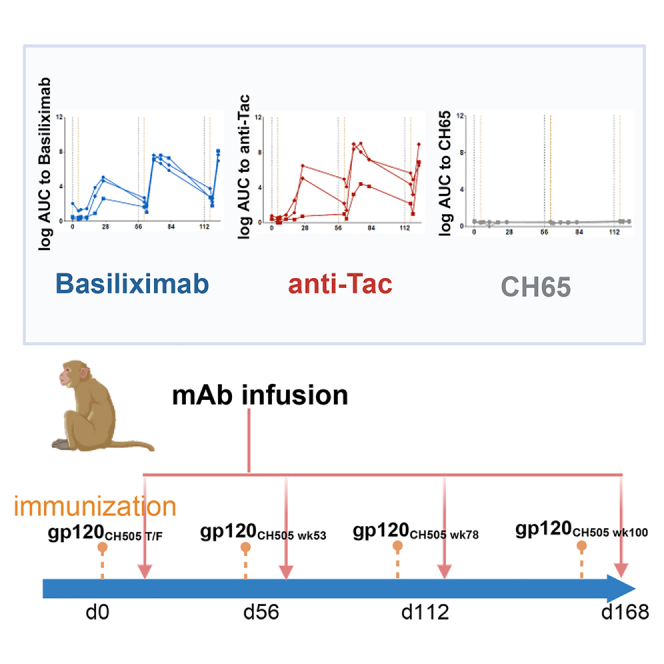

## Introduction

Human immunodeficiency virus-1 (HIV-1) infection results in an increasing, hierarchical loss of CD4^+^ T cells and chronic immune activation, culminating in progressive immune dysfunction.^1^^,^^2^ After nearly four decades of effort, an effective HIV-1 vaccine has yet to be developed.^3^ Many current candidates are designed to elicit broadly neutralizing antibodies (bnAbs) that would be able to prevent infection by the highly variable HIV-1, but reliable elicitation of bnAbs has proven elusive. Anti-HIV-1 bnAbs are characterized by one or more unusual traits: poly- or auto-reactivity for host antigens; long variable heavy-chain complementarity-determining region 3 (HCDR3) loops, and/or high levels of somatic hypermutation.^4^^,^^5^^,^^6^ These bnAb characteristics are disfavored by the immune system and regulated by tolerance mechanisms^7^; for example, maturation of CD4 binding-site (CD4bs) Abs is limited by immune tolerance in bnAb germline knock-in mice.^8^ One strategy to overcome these tolerance checkpoints would be the development of unconventional and rational vaccine design strategies to transiently release immune tolerance to expand and potentiate the bnAb precursor pool.

Upon vaccination or pathogen exposure, antigen-specific CD4^+^ and CD8^+^ T cells are primed in the T cell zone of secondary lymphoid tissues, and then differentiate into follicular helper T (Tfh, ICOS^+^ PD-1^+^ CXCR5^+^ CD4^+^) cells and follicular cytotoxic T (Tfc, CXCR5^+^ CD8^+^) cells. Tfh and Tfc become part of efficient immune response niches within B cell follicles (i.e., germinal centers [GCs]) that promote the development of protective antibody responses; meanwhile, regulatory CD4^+^ T cell populations such as follicular regulatory T (Tfr, FoxP3^+^ CXCR5^+^ CD4^+^) cells control the overall magnitude of the GC reaction by limiting Tfh cells and regulating GC B cells.^9^^,^^10^^,^^11^^,^^12^^,^^13^^,^^14^ Interestingly, we previously showed that people chronically infected with HIV-1 who had generated bnAbs had a lower frequency of circulating regulatory T (Treg) cells compared to HIV-1-infected persons who developed little or no antibody neutralization breadth; we also observed increased expression of PD-1 on both Treg cells and Tfr cells in the bnAb group, suggesting that reduced peripheral immune tolerance was permissive for the development of bnAbs.^6^^,^^15^ Loss or functional impairment of Treg cells is associated with autoantibody production in patients with autoimmune diseases^16^^,^^17^^,^^18^; taken together, these findings suggest that regulatory CD4^+^ Treg cells may constrain the production of HIV-1 bnAbs during infection and may pose a barrier to bnAb development after vaccination. *In vitro* modulation of inhibitory pathways has been shown to restore some functional cellular and humoral responses in persistent viral infection^19^^,^^20^^,^^21^; thus, we postulated that transient manipulation of the GC response by disrupting Treg activity to release peripheral tolerance might permit otherwise disfavored B cell maturation after vaccination and allow for the development of bnAbs.

CD25, the α-chain of the interleukin-2 receptor (IL-2RA) is a pivotal surface marker in Treg cells, and is essential for high-affinity IL-2 binding to activate and maintain cell differentiation and proliferation.^22^ CD4^+^ T cells with constitutive CD25 expression are enriched for Treg cell activity and help maintain immune tolerance and homeostasis.^16^^,^^23^^,^^24^^,^^25^^,^^26^^,^^27^ IL-2 inhibits the differentiation of Tfh cells and controls GC formation and long-lived antibody responses.^28^ Moreover, IL-2-derived signaling pathways promote Treg survival, as CD25 downregulation of activated Tregs in non-lymphoid tissues leads to apoptosis.^29^ Clinically, this has been exploited by monoclonal antibody (mAb) drugs that alter the immune landscape in transplant recipients. IL-2 inhibitor basiliximab permanently blocks CD25 and is used to prevent graft-versus-host disease after transplantation.^30^ Daclizumab (anti-Tac) is an antibody that binds to CD25 and mediates Treg depletion; this drug was used for the prevention of graft-vs-host disease and the treatment of relapsing multiple sclerosis prior to being withdrawn from the market in 2018 due to adverse events.^31^ While it is known that these drugs alter the immune response in these clinical settings, systematic study of vaccine response in the presence of these drugs is lacking.

B cell lineage vaccine strategies based on the isolation of bnAbs and sequential envelope (Env) gp120s from an HIV-1 infected individual have been designed to mimic the coevolutionary events that drove affinity maturation and bnAb development.^5^^,^^32^^,^^33^^,^^34^ In this study, we immunized a cohort of rhesus macaques (RMs) with a sequential HIV-1 Env gp120 vaccine strategy that had been previously shown to initiate CD4 binding site (CD4bs) antibody lineages but not bring them to full maturity^8^ and treated the RMs with basiliximab, rhesusized anti-Tac, or an irrelevant control mAb (CH65^35^) to determine if relaxation of immune tolerance would permit the development of HIV-1 bnAbs.

## Results

### Treg perturbation strategies elicit a similar plasma antibody response to HIV-1 Envs

**Figure 1:**
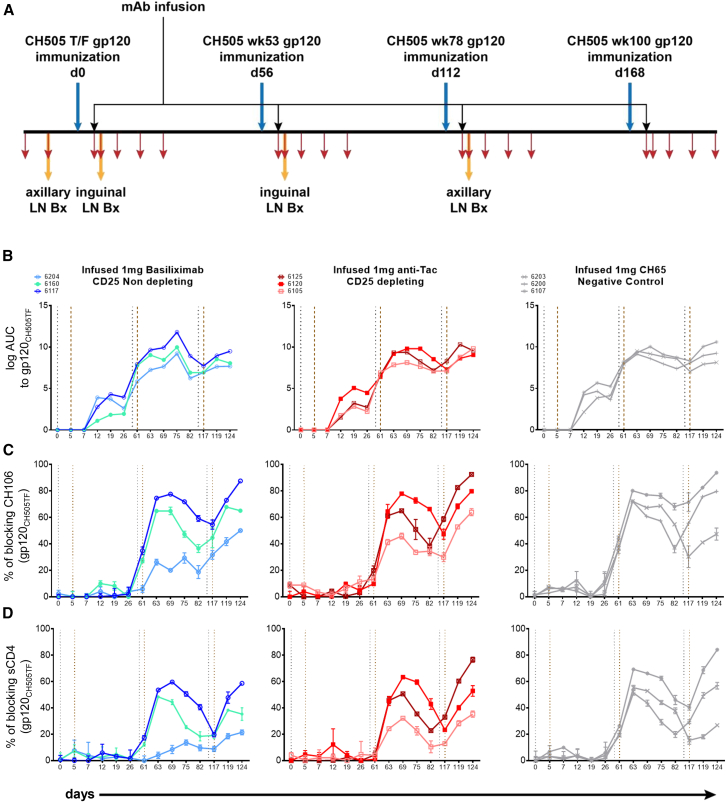
Immunization, treatment, sampling strategy, and plasma antibody response (A) Rhesus macaques were vaccinated with sequential HIV-1 gp120 on days 0, 56, 112, and 168 (blue arrows) with the same strategy. Anti-IL2RA or control mAbs were infused on the 5th day after each vaccination (black arrows). Lymph node samples were taken on days −9 (pre-immunization), 7, 63, and 119 (7 days after the first three immunization) and processed for single memory B cell sorting and mAb isolation (yellow arrows). Peripheral blood was collected as shown by red arrows (days −16, days of mAb infusion and lymph node excision, and subsequently once per week for three consecutive weeks after each lymph node collection). Plasma was tested for binding to HIV-1 Env gp120_CH505TF_ (B), blocking of CH106 binding (C) and blocking of sCD4 binding (D). RMs treated with basiliximab (left), anti-Tac (middle), and control CH65 (right) mAbs are shown, respectively. Vertical gray dotted lines indicate immunization time points, brown lines indicate mAb infusions.

Nine male, adult RMs randomly divided into three groups (3 per group) were immunized with sequential clade C HIV-1 CH505 Env gp120 proteins (CH505 TF, CH505wk53, CH505wk78, and CH505wk100) that were originally isolated from a person recruited into the CHAVI 001 cohort about 4 weeks after infection and who was followed for over three years^8^^,^^33^^,^^34^ (Figure 1A). Five days after each Env gp120 vaccination (days 0, 56, 112, and 168), the RMs were infused with mAbs to modulate immune tolerance. Group 1 was given 1 mg of basiliximab to permanently block the IL-2 receptor α-chain, group 2 was given 1 mg of rhesusized anti-Tac to deplete Tregs, and group 3 was given 1 mg of rhesusized influenza antibody CH65 as a control. We collected peripheral blood and lymph node samples as shown (Figures 1A and S1A).

We observed that the plasma antibody binding titers to gp120_CH505TF_ increased 2-fold after the second immunization in all RMs, regardless of mAb treatment group, compared to titers observed after the first immunization (Figure 1B). We assessed the development of CD4bs antibodies by three methods. Deletion of the amino acid at position 371 of gp120_CH505TF_ abrogates binding of CD4bs bnAbs^36^; we did not find evidence of differential binding of RM plasma to the gp120_CH505TF_ over the Δ371 mutant (Figures S1B and S1C) suggesting that antibodies were not primarily directed to that epitope. We also used resurfaced stabilized core 3 (RSC3) and knockout mutant ΔRSC3^37^ which identify CD4bs antibodies and found no evidence for differential binding (Figures S1D–S1F). We tested for plasma antibody blocking of the binding of CD4bs bnAb CH106 to gp120 Envs gp120_CH505TF_ (Figure 1C) and heterologous gp120_63521_ (Figure S1G); all RMs developed antibodies that blocked CH106 binding, though that activity differed among RMs. Similarly, we tested for plasma antibody blocking of soluble CD4 (sCD4) binding to Env gp120_CH505__T__F_ (Figure 1D) and gp120_63521_ (Figure S1H); the pattern of blocking was similar to that for CH106 but of lower magnitude. Overall, the data suggested that vaccination elicited CD4bs antibodies but not VRC01-class bnAbs and that administration of anti-CD25 treatment did not alter that response.

**Figure 2:**
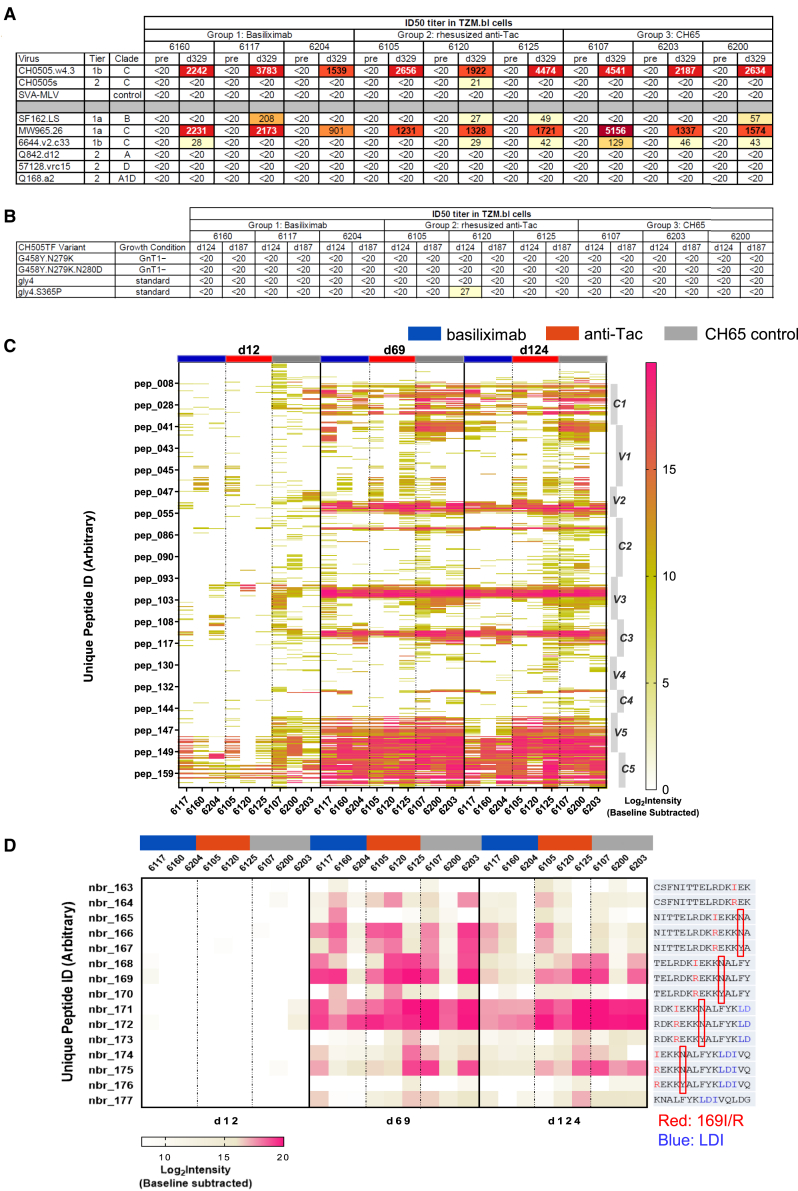
Neutralization and linear epitope mapping of plasma antibodies (A–D) Plasma samples collected pre-immunization and on day 329 were heat inactivated before being tested against a series of pseudotyped viruses from a small breadth panel (A) and a panel of variants designed to identify bnAb precursors (B). ID50 titer is shown. (C) RM plasma samples from days 12, 69, and 124 were tested for binding to overlapping linear Env peptides from HIV consensus and vaccine strains, 59 sequences for CH505 antigens including CH505TF. Data plotted are baseline-substracted Log_2_ Intensity (Post/Pre) values for binding to all peptides for the 59 CH505 sequences in the library. Peptide numbers are aligned to Con M sequences. Amino acid positions (based on HXB2 numbers) are each peptide number and the peptide sequences for Con M and CH505TF are given in Table S1. HIV-1 Env regions are shown on the right of the heatmap, sample time points are on the top. RMs treated with basiliximab (left), anti-Tac (middle), and control CH65 (right) mAbs are shown in blue, red, and gray, respectively. Amino acids in dominant B cell epitopes in V2 are shown in (D). V2 position is shown on the left and peptide sequence is shown on the right. Position N173Y is highlighted by red boxes.

We tested for neutralization using the TZM-bl assay on serum samples from before immunization and at days 124, 187, and 329. All RMs neutralized the tier 1 clade C autologous CH0505w4.3 strain and heterologous MW965.26 strain on day 124 and later samples (Figures 2A and S2A). Some RMs developed neutralizing antibodies against the tier 1 clade B SF162.LS strain but the titers for most animals were <1:100. None of the RMs developed antibodies that could neutralize any tier 2 virus tested including the autologous CH0505s strain (Figures 2A and S2A). Because early precursors of bnAb lineages do not always show potent or broad neutralization, we also tested for the initiation of bnAb lineages using mutated versions of the CH505TF strain. Samples from these RMs did not neutralize the G458Y.N279K variant or related variants (Figure 2B) suggesting there was not induction of the CH235 bnAb lineage^38^; similarly, there was no neutralization of gly4 variants (Figure 2B) suggesting that there was not induction of the CH103 bnAb lineage.^38^ Collectively, these data indicate that the sequential immunization strategy coupled with anti-CD25 treatment was insufficient to start bnAb lineages in these RMs.

### Mapping of HIV-1 Env-induced B cell linear epitopes

This vaccine strategy was able to elicit CD4bs antibodies but not bnAbs or bnAb precursors; it is possible that relaxed tolerance might have led to the initiation of antibodies to epitopes shown to be important such as glycan-containing regions on the surface of gp120, variable regions 1 and 2 (V1/V2) and glycan-associated C3/V3 regions.^39^ To assess this, we used a linear epitope mapping peptide microarray covering the full length of consensus gp120 Env from vaccine strains including A244, TH023, MN, C.1086, C.TV1, C.ZM651, and 59 sequences for various CH505 antigens including CH505_TF_. Plasmas from one week after each mAb treatment (days 12, 69, and 124) were tested (Figures 2C and S2B). By day 12, samples from control RMs bound to more B cell linear epitopes than anti-CD25 treated groups, especially in the V1/V2 and C3/V3 regions. After the 2^nd^ and 3^rd^ immunizations and mAb treatments, binding to HIV-1 Env-specific linear epitopes evened out among the groups, although we observed less binding to some epitopes for samples from RMs that received anti-CD25 treatment (Figure 2C) (e.g., C1- group median binding was positive for 169 and 170 C1 peptides [including 31 overlapping peptides across 14 Env strains] at D69 and D124 for anti-CD25 group, respectively, compared to 291 and 267 peptides for the control group). Binding to the V2 was similar among RMs in all groups after the second and third immunizations (Figure 2C), and strong reactivity to the V2 has been observed for other vaccine strategies in RMs.^40^ We found evidence of an epitope specific response centered at position 173; peptides nbr_171 and nbr_172 have an asparagine (N) at position 173 and showed strong binding while nbr_173 has a tyrosine (Y) at that position and showed no binding (Figure 2D). These results indicate that N173 was required by antibodies recognizing the CH505 V2 epitope in all CH505 sequentially immunized RMs.

### Effect of transient perturbation of Tregs on T cell populations

**Figure 3:**
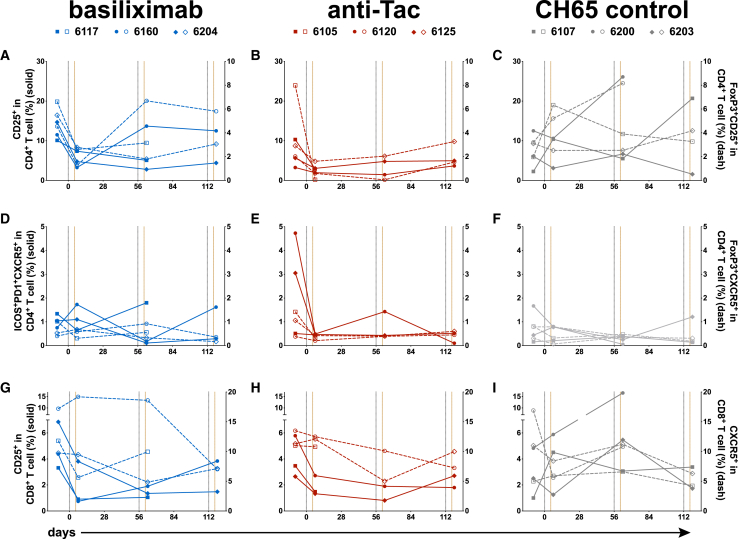
Effect of anti-CD25 treatment on germinal center populations in lymph nodes (A–I) Lymph nodes taken from RMs at a series of time points were analyzed. Shown is the proportion of CD25^+^ CD4^+^ T (A, B, and C, solid lines) and CD8^+^ T (G, H, and I, solid lines) cells, percentage of regulatory T (Treg, FoxP3^+^ CD25^+^ CD4^+^, [A, B, and C], dashed lines), follicular helper T (Tfh, ICOS^+^ PD-1^+^ CXCR5^+^ CD4^+^, [D, E, and F], solid lines), follicular regulatory T (Tfr, FoxP3^+^ CXCR5^+^ CD4^+^, DEF, dashed lines), and follicular cytotoxic T (Tfc, CXCR5^+^ CD8^+^, [G, H, and I], dashed lines) cells in lymph nodes. Vertical gray dotted lines indicate immunization time points, brown lines indicate mAb infusions.

To assess the efficacy of anti-CD25 treatment, we phenotyped immune cells from lymph nodes after mAb administration; the gating strategy for Treg/Tfh/Tfr/Tfc is shown in Figure S3A. Due to sample limitations, not all RMs have data for all time points. As expected, we found a decreased frequency of CD25^+^ CD4^+^ T cells in lymph nodes after the first anti-CD25 mAb infusion (mean: 12.1% vs. 5.13% in basiliximab group; mean: 6.4% vs. 2.64% in anti-Tac group; Figures 3A and 3B) while the CH65 mAb control did not have a consistent effect for any of the three infusion time points studied (Figure 3C). This effect of anti-CD25 mAb infusion was observed for both total CD25^+^ CD4^+^ T cells and FoxP3^+^ CD25^+^ CD4^+^ T cells (Tregs). The second infusion of anti-Tac maintained the low frequency of CD25^+^ CD4^+^ T cells (Figure 3B) while basiliximab infusion gave more variable results (Figure 3A), In contrast, FoxP3^+^ CXCR5^+^ CD4^+^ Tfrs, which play an important role in limiting GC responses, were similar in all three groups (Figures 3D–3F), as were ICOS^+^ PD-1^+^ CXCR5^+^ CD4^+^ Tfhs (Figures 3D–3F). We found a decreased frequency of CD25^+^ CD8^+^ T cells after the first anti-CD25 mAb treatment (mean: 4.85% vs. 1.82% in basiliximab group; mean: 3.97% vs. 1.84% in anti-Tac group; Figures 3G and 3H) like what we observed with CD25^+^ CD4^+^ T cells, while CXCR5^+^ CD8^+^ Tfcs were similar in all three groups (Figures 3G–3I). We further characterized the expression level of multiple activation and subset markers following mAb treatment (Figure S3B). Given the sparse sampling, we cannot draw firm conclusions from these data, but we did find a slight increase of CD95 expression in lymph node CD8^+^ T cells (mean: 93.3% vs. 94.67% in basiliximab group; mean: 89.97% vs. 92.97% in anti-Tac group) after the first anti-CD25 infusion, though this effect was less on Tfcs (Figure S3B).

Total T cells can be divided into four distinct subsets, central memory T (T_CM_, CCR7^+^ CD45RA^−^), effector memory T (T_EM_, CCR7^−^ CD45RA^−^), naive T (T_NC_, CCR7^+^ CD45RA^+^), and terminally differentiated effector memory T cells (T_EMRA_, CCR7^−^ CD45RA^+^); the gating strategy is shown in Figure S3C. These subsets have been shown to have different effector functions and proliferative capacity, thus alterations due to anti-CD25 treatment might lead to different outcomes. In contrast to the CD25^+^ subsets describe previously, we observed no consistent alterations in these four T cell subsets (Figure S4).

We also examined the frequencies of T cell subsets in peripheral blood mononuclear cells (PBMCs) before and after the first mAb infusion. Like the results from lymph nodes, anti-CD25 treatment reduced the frequency of CD25^+^ CD4^+^ T cells, but the magnitude of the change was less (Figure S3D); overall, no major alteration of peripheral blood T cells was detected.

Taken together, anti-CD25 mAb treatment effectively perturbed CD25^+^ T cell populations, particularly Tregs, but these effects were predominantly observed after the first infusion, which may highlight the importance of treatment timing in relation to vaccination or other immune interventions.

### Effect of transient perturbation of Tregs on B cell populations

We then assessed B cell subsets using the gating strategy shown in Figure S5A. Like what we observed for PBMC T cells, there was no consistent pattern of alteration of B cell subsets, although non-class-switched memory B (IgD^+^ CD27^+^ CD20^+^) decreased in anti-CD25 treated groups compared with CH65 treated RMs (Figure S5B). Similarly, the frequencies of naive B (IgD^+^ CD27^−^ CD20^+^, mean: 34.87% vs. 26.17% in basiliximab group; mean:35.37% vs. 31.67% in anti-Tac group) and transitional B (IgD^+^ IgM^hi^ CD27^−^ CD20^+^, mean: 29.67% vs. 20.37% in basiliximab group; mean: 32.63% vs. 31.87% in anti-Tac group) cells were decreased in anti-CD25 treated RMs compared to the CH65 treated control animals (Figure S5B), though small group sizes precludes statistical analysis.

**Figure 4:**
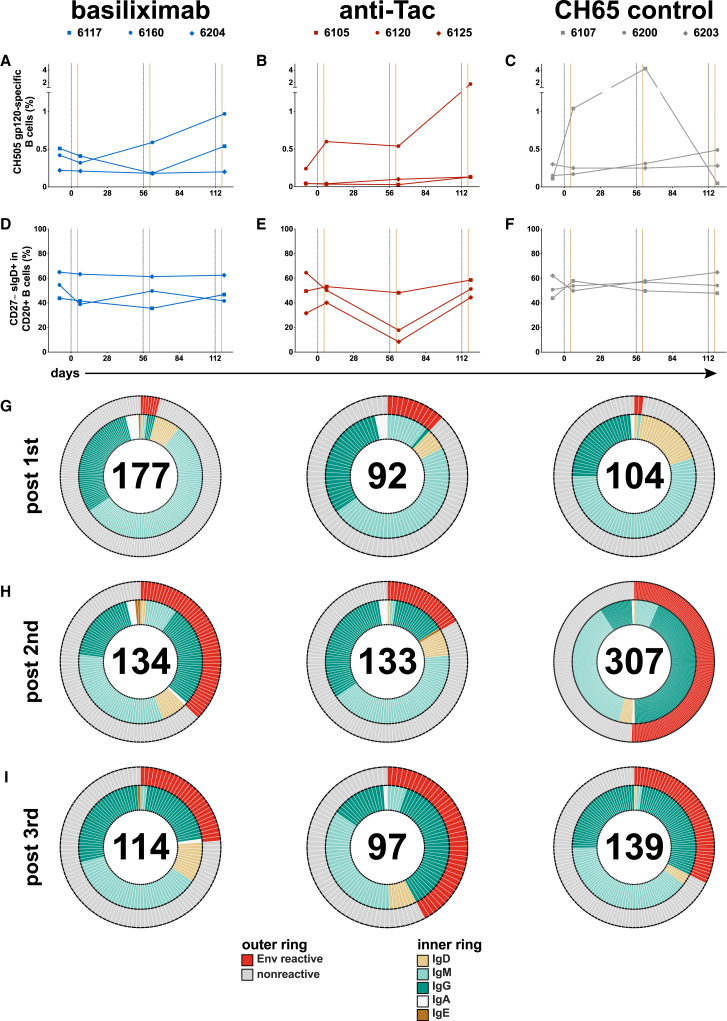
Antibody repertoire in vaccinated RMs Percentage of CH505 gp120-specific B cells (A, B, and C) and naive B cells (D, E, and F) is shown. Isolated mAbs from lymph node samples after the 1st (day 7, G), 2nd (day 63, H), and 3rd (day 119, I) immunizations are shown. HIV-1 Env reactivity of isolated mAbs (outer ring) is shown in red if the screening optical density ≥0.25, reactivity below that cutoff is shown in gray. The inner ring shows isolated antibody isotype. The numbers of isolated mAbs are in the middle of the pie charts.

To facilitate antibody repertoire analysis, we examined lymph node B cell populations in the context of sorting antigen-specific B cells using dual staining with fluorochrome-conjugated gp120_CH505_. The sorting panel did not include surface IgM staining, thus, not all the same subsets could be examined; the gating strategy is shown in Figure S5C. The frequency of B cells that bound CH505 gp120 hook is shown in Figures 4A–4C, and we found the frequency of antigen-specific B cells is like what has been observed in other studies,^40^ including the higher frequency of cells in some samples. We did not see a consistent pattern of change in either CD27^+^ CD20^+^ B cells (Figure S5D) or non-class-switched memory B cells (Figure S5E), but we did observe that two anti-Tac treated RMs showed a reduced frequency of naive B cells after the second treatment (Figures 4D–4F).

### HIV-1 Env-specific memory B cell repertoire after anti-CD25 administration

We isolated 544 mAbs from basiliximab-treated RMs, 403 mAbs from anti-Tac-treated RMs, and 591 mAbs from CH65-treated control RMs (Table 1; Figures 4G–4I). A small number of pre-immunization mAbs from basiliximab and anti-Tac treated RMs were HIV-1 Env reactive, as determined by ELISA; many of these were IgM and weakly reactive (Figure S6A). A small number of Env-reactive mAbs were isolated after the first immunization (Figure 4G); most of these were IgM with a smaller number of IgD and IgG mAbs. After the second immunization with gp120_CH505wk53_, we recovered a larger number of HIV-1 Env-reactive antibodies in all groups (Figure 4H), however, those in the anti-Tac group were less numerous (22/133, 16.5%) than in the basiliximab and CH65 control treated groups (50/134, 37.3% and 155/307, 50.5%, respectively). After the 3^rd^ immunization, recovery of Env-reactive mAbs was similar, with an increase in recovery for the anti-Tac group (Figure 4I) (basiliximab: 27/114, 23.7%; anti-Tac: 41/97, 42.2%; CH65: 45/139, 32.4%).

**Table 1:** Env-reactivity of isolated mAbs from pre-vaccine, post-1st, 2nd, and 3rd immunization in basiliximab, anti-Tac, and CH65-treated groups

Treatment group	Animal ID		pre-vaccine	post 1st	post 2nd	post 3rd
basiliximab	6117	mAbs total	59	72	85	20
		Env reactive	12	5	39	3
	6160	mAbs total	24	44	29	63
		Env reactive	0	0	7	23
	6204	mAbs total	36	61	20	31
		Env reactive	0	2	4	1
anti-Tac	6105	mAbs total	33	30	85	62
		Env reactive	6	8	17	19
	6120	mAbs total	0	0	0	0
		Env reactive	0	0	0	0
	6125	mAbs total	48	62	48	35
		Env reactive	2	3	5	22
CH65	6107	mAbs total	6	57	141	10
		Env reactive	0	1	120	0
	6200	mAbs total	12	47	49	63
		Env reactive	0	1	7	29
	6203	mAbs total	23	0	117	66
		Env reactive	0	0	28	16

**Figure 5:**
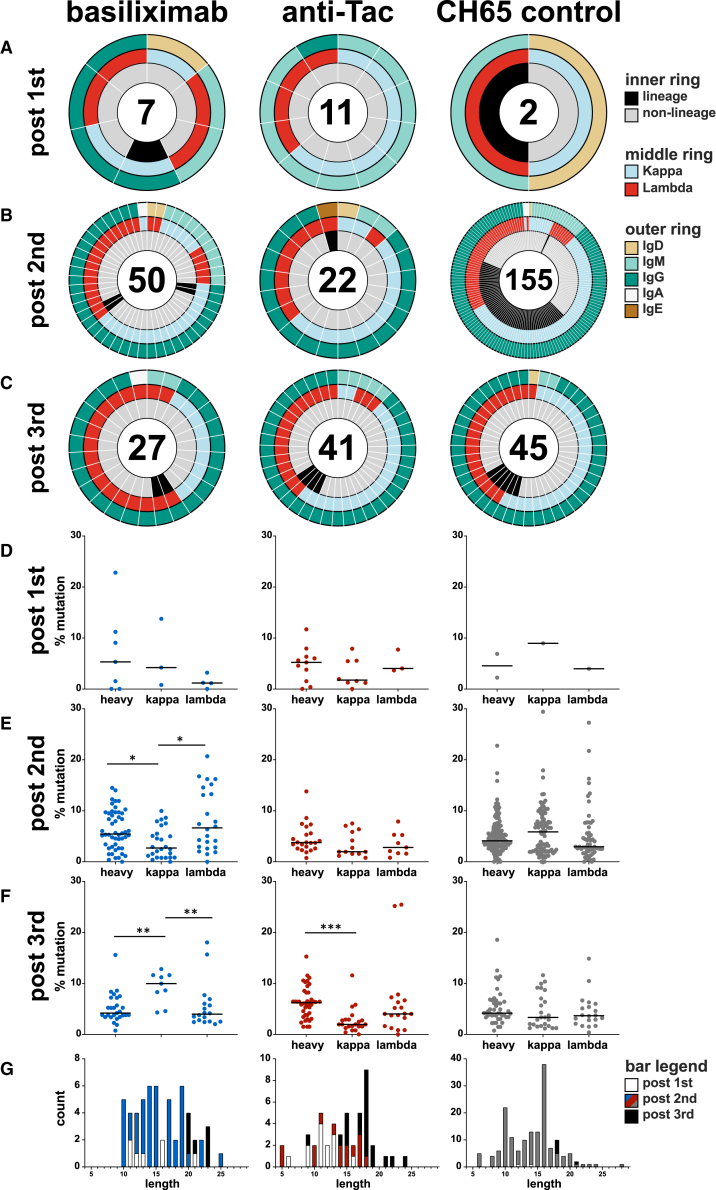
Characteristics of Env-reactive mAbs Env-reactive mAbs isolated after the 1st, 2nd, and 3rd immunizations are shown (A, B, and C). Clonal lineages were identified as antibodies using the same V_H_, J_H_, V_κ/λ_ and J_κ/λ_ gene usage and heavy/light chain CDR3 length; lineage membership was confirmed by analysis using Cloanalyst. The inner ring denotes clonal lineage membership; the middle ring shows kappa/lambda usage; the outer ring shows antibody isotype. Heavy/kappa/lambda chain mutation frequency of Env-reactive mAbs isolated after the 1st, 2nd, and 3rd immunizations is shown for basiliximab (D), anti-Tac (E), and CH65 (F) groups. HCDR3 length of Env-reactive mAbs from each group are shown in (G). (D–F) Kruskal-Wallis H test and Dunn’s multiple comparisons test. ^∗^*p* < 0.05, ^∗∗^*p* < 0.01, ^∗∗∗^*p* < 0.001. CDR3, complementarity-determining region 3; HC, heavy chain; J_H/κ/λ_, joining region of heavy/kappa/lambda chain; V_H/κ/λ_, variable region of heavy/kappa/lambda chain.

As expected, few Env-reactive mAbs were recovered after the first immunization and there were no obvious patterns of isotype or light chain usage (Figure 5A). Recovery after the second and third immunizations was more robust; the majority was IgG (Figures 5B and 5C) with a slight predominance of kappa chain usage except for the basiliximab group after the third immunization (Figure 5C). Analysis identified mAbs that were clonally related (i.e., that derived from the same parental B cell); we observed a few clonally related mAbs after the second and third immunizations for the anti-CD25-treated groups, while a much larger number of mAbs isolated from the CH65 control group after the second immunization were clonally related (Figure 5B, 69/155, 44.5%); this higher proportion of clonally related mAbs is typical of a vaccine recall response in humans^41^ and RMs.^15^ There were fewer clonally related mAbs isolated in the CH65 control group after the third immunization (6/45, 13.3%), but this may also be due to sampling non-draining axillary lymph nodes at that time point. Despite the apparent stability in the frequencies of GC immune cell subsets, the reduced of percentage of clonally related clones suggests that the GC response was nonetheless altered following anti-CD25 administration. Thus, anti-CD25 treatment may have modulated the GC environment leading to a larger number of small clonal lineages and/or singletons and fewer clonal expansions.

### Treg disruption and development of autoreactive HIV-1 Env-specific antibodies in lymphoid tissues

HIV-1 bnAbs usually have one or more of three characteristics: high degrees of somatic mutation, long HCDR3 loop length, and/or autoreactivity.^6^ The mutation frequencies of recovered mAbs demonstrated modest levels of somatic mutation for heavy and light chains (Figures 5D–5F) with no obvious patterns; mutation frequencies were slightly lower than observed in other vaccine studies in RMs.^40^^,^^42^^,^^43^ The few mAbs with high degrees of mutation did not display breadth of binding (Figure S6B). Similarly, observed HCDR3 lengths of isolated Env-reactive mAbs (Figure 5G) were similar to that seen for vaccine responses in humans^41^^,^^44^ and RMs.^40^ We did isolate one mAb with HCDR3 of 28 amino acids from the CH65 control group and two from the basiliximab group with HCDR3 of 25, and these displayed similar binding breadth as those with short HCDR3 (Figure S6C).

Loss or functional impairment of Tregs is associated with autoantibody production in patients with autoimmune diseases. Since we observed a degree of T cell perturbation following anti-CD25 treatment (Figure 3), we sought to determine if plasma antibody or recovered mAbs displayed autoreactivity. We screened plasma and purified plasma IgG against 9 autoantigens: double stranded DNA (dsDNA), centromere B, histones, histidine-tRNA ligase (Jo1), Sjögren’s-syndrome-related antigen A (SSA) and B (SSB), topoisomerase I (Scl-70), Smith antigen (Sm), and ribonuclear protein (RNP). We tested for auto-antibodies in plasma and using purified plasma IgG (Figure S7A); the treatment and infusion schedule was not associated with the development of plasma autoantibodies, but one RM in the basiliximab group was found to have baseline Scl-70 reactivity that did not change over the course of the study.

**Figure 6:**
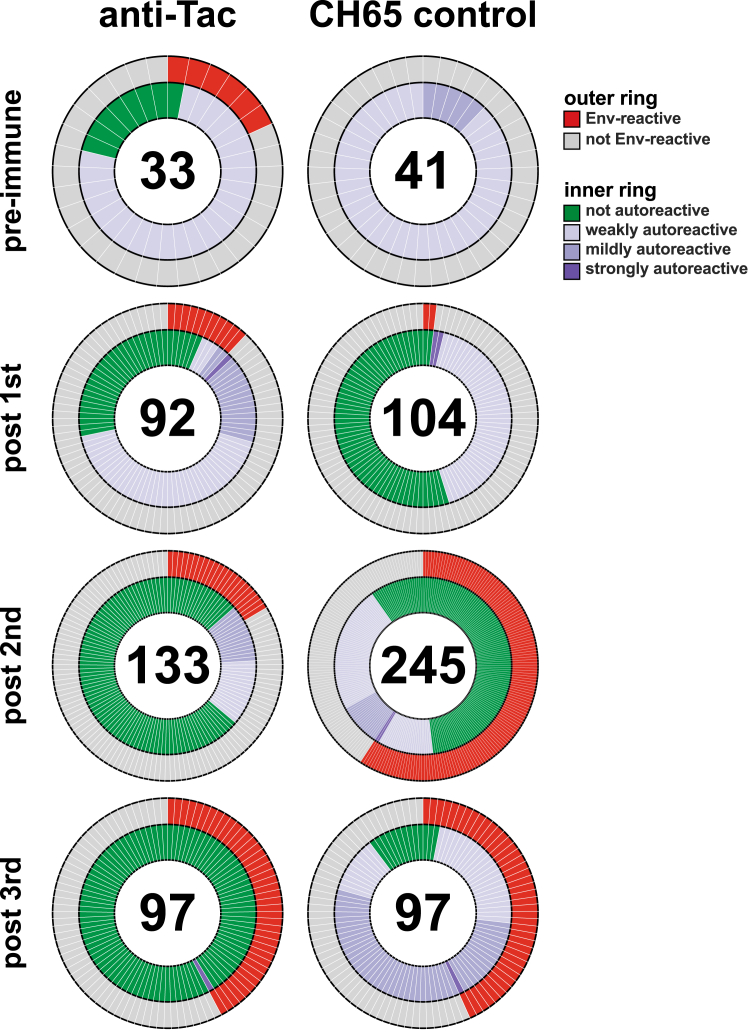
Autoreactivity of isolated mAbs from anti-Tac-treated and CH65-treated RMs Isolated mAbs were tested for reactivity to human autoantigens: double-stranded deoxyribonucleic acid (dsDNA), centromere B, histones, histidine-tRNA ligase (Jo1), Sjögren’s syndrome-related antigen A (SSA) and B (SSB), topoisomerase I (Scl-70), Smith antigen (Sm), ribonucleoprotein (RNP). Positivity thresholds for binding to Env proteins were set at 0.25 AU; thresholds for autoantigens were >0.75 AU for strongly autoreactive, 0.5–0.75 AU for mildly autoreactive, and 0.25–0.5 AU for weakly autoreactive. The numbers of tested mAbs are in the middle of the pie chart. Isolated mAbs from basiliximab-treated animals were not screened.

It is possible that T cell perturbation could lead to polyreactivity in individual B cells that does not result in plasma activity; thus, we tested 842 isolated mAbs from the anti-Tac and CH65 control groups using the human autoantigen panel (Figure 6). The majority of the Env-reactive mAbs from both groups were not autoreactive or displayed mild degrees of autoreactivity; the exception was a higher proportion of autoreactive antibodies observed after the third immunization in the CH65 control group. In contrast, after the third immunization in the anti-Tac group, only a single Env-reactive antibody was autoreactive. This result was opposite of what we expected given the T cell perturbation data (Figure 3), leading us to hypothesize that the infused antibodies may have only been present and active transiently after infusion.

### Kinetics of infused antibodies

**Figure 7:**
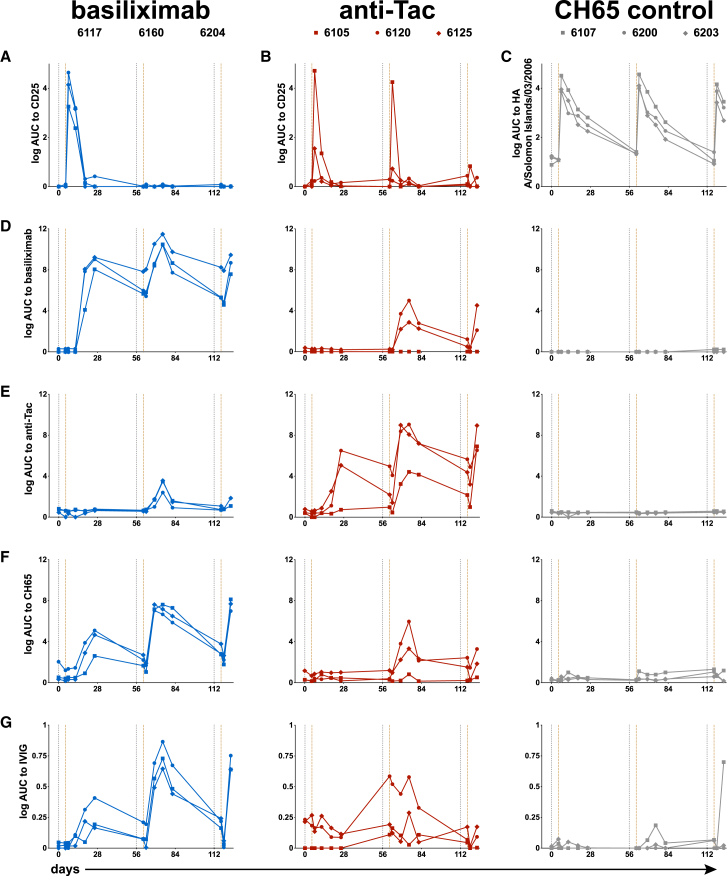
Infused antibody kinetics (A–C) RMs infused with anti-human IL2RA mAbs were tested for plasma antibody reactivity against human CD25 protein. RMs infused with influenza mAb CH65 were tested for binding to hemagglutinin from A/Solomon Islands/3/2006 (H1N1) (C). (D–F) Anti-infused mAb antibody response in plasma. (G) Rheumatoid factor-IgM antibodies were tested in plasma. RMs plasma collection days shown on *x* axis. Vertical gray dashed lines indicate immunizations, brown dashed lines indicate mAb infusion time points. Binding activity (log AUC) shown on *y* axis. Basiliximab, anti-Tac, and CH65 control mAb treated subjects from each group were presented in blue, red, and gray. AUC, area under the curve; mAb, monoclonal antibody; IVIG, intravenous immune globulin; RMs, rhesus macaques.

We tested plasma samples across the study for binding to the targets of the infused mAbs: human CD25 (Figures 7A and 7B) and influenza hemagglutinin from A/Solomon Islands/03/2006 (H1N1) (Figure 7C). The kinetics of CH65 infusion were as expected with a rapid rise and decay consistent with first-order kinetics (Figure 7C). In contrast, anti-CD25 activity was only detected after the first infusion of basiliximab and was rapidly cleared in the first few weeks after infusion (Figure 7A). Anti-CD25 activity in the anti-Tac group showed a similar early peak and rapid clearance, except for RM 6120 which did not show the expected rise (Figure 7B). RM 6105 showed transient anti-CD25 activity after the second infusion with rapid clearance, and no animals had significant activity after the third infusion. These data demonstrate that the infusion of antibodies against CD25 resulted in rapid clearance.

We examined for antibodies against the infused mAbs to determine if rapid clearance was due to the presence of anti-drug antibodies. RMs infused with basiliximab all made antibodies against the drug which were boosted after subsequent infusions (Figure 7D); two RMs given anti-Tac also developed low level binding to basiliximab while no RMs in the CH65 group showed similar activity (Figure 7D). RMs given anti-Tac developed antibodies against that antibody, although this was not as consistent as in the basiliximab group (Figure 7E); a low level of anti-Tac antibodies was detected in the basiliximab group after the second infusion and no RMs in the CH65 group developed that activity (Figure 7E). Infusion of CH65 was not associated with the development of anti-drug antibodies (Figure 7F), but the basiliximab and anti-Tac groups did develop antibodies against CH65 (Figure 7F).

These data suggest that anti-CD25 treatment may have resulted in antibodies against IgG, also known as rheumatoid factor.^45^ To test this, we examined binding of rhesus plasma IgM to human intravenous immune globulin (IVIG). The basiliximab infused RMs developed detectable IgM binding to IVIG that was boosted by later infusions (Figure 7G). The anti-Tac infused RMs also developed IgM binding to IVIG, although this was less consistent. Interestingly, two RMs in this group had detectable activity at baseline (Figure 7G), including RM 6120 which showed no detectable anti-CD25 activity after infusion (Figure 7B). One RM in the CH65 group developed detectable IgM activity that was boosted after the third infusion (Figure 7G), though this was not associated with altered kinetics (Figure 7C). Taken together, these data indicate that infusion of anti-CD25 mAbs was associated with a break in tolerance resulting in detectable anti-drug antibodies and IgM anti-IgG antibodies that may have limited their utility in altering the anti-Env response to vaccination.

## Discussion

Despite nearly four decades of work, no broadly effective preventative HIV-1 vaccine has been developed. The characteristics of HIV-1 bnAbs are attractive targets for vaccine design, but HIV-1 bnAbs share characteristics indicative of regulation by immune tolerance,^6^ and we have previously shown that chronically HIV-1-infected individuals with plasma bnAbs have immune perturbations consistent with altered tolerance, including a lower frequency of Treg cells and a higher PD-1 expression on Treg cells.^15^ Previous work showed that the acquisition of bnAb activity can be associated with autoreactivity, and some HIV-1 bnAbs and systemic lupus erythematosus (SLE)-associated autoantibodies can arise from similar pools of B cells,^33^^,^^46^^,^^47^ suggesting a mechanistic link between bnAb development and autoimmunity. We hypothesized that transient manipulation of Treg populations might be permissive for the initiation of bnAb B cell lineages. This small study was designed to test whether Treg manipulation during the GC response, 5 days after vaccination, would allow further maturation of the B cell response to a sequential CH505 gp120 Env immunization regimen that was designed to guide the development of CD4-binding site bnAbs.

The Treg modulating drugs chosen for this study, basiliximab and anti-Tac (daclizumab) have been used for the treatment of some kinds of autoimmunity and to combat graft-versus-host disease^30^^,^^31^; their ready availability made them practical to test as HIV-1 vaccine adjuvants. Basiliximab has been widely used to reduce rates of acute rejection at the time of transplantation, while daclizumab was also used for that purpose but was withdrawn in 2018 because of serious side effects including autoimmune encephalitis. Given their potent effects on the immune system, these drugs would be impractical and likely dangerous to use to persistently alter Treg populations among vaccinees to permit bnAb development. Tregs play key roles in controlling immune responses and can actively suppress self-reactive inflammatory T cell responses and autoimmunity.^48^ Anti-CD25 mAb drugs have been associated with the development of autoimmunity, immunopathology, and lymphoproliferative disease.^49^^,^^50^ We designed this study to test whether a short exposure could have a beneficial effect, and if successful, we anticipated that future, more targeted and safer adjuvant approaches could be developed that did not involve whole body Treg manipulation.

Anti-CD25 treatment did not diminish or enhance the response to the CH505 regimen (Figures 1 and 2) though we did observe reduction of Treg populations in biopsied lymph nodes (Figure 3). Broadly neutralizing antibodies were not elicited (Figures 2A and S2A) and we did not detect the initiation of bnAb lineages using neutralization assays designed for that purpose (Figure 2B). We did not detect skewing of the Env-specific B cell response (Figures 4G–4I and 5A–5C) and the mutation frequencies and HCDR3 lengths were similar regardless of treatment group (Figures 5E–5G). We did find that clonal lineage expansion was recovered less from anti-CD25 treated RMs after the second immunization (Figure 5B), but that effect was not detected after the third immunization (Figure 5D); it is possible that our detection of clonal lineage expansion was limited by sampling non-draining lymph nodes after the third immunization (Figure 1A). Further examination of this observation will have to be done in future studies, but lower recovery of clonally related B cells is consistent with prior observations of lineage distributions in other RM experiments.^40^

Although no bnAb lineages were elicited, it was possible that manipulation of Tregs resulted in greater degrees of autoreactivity and/or polyreactivity; thus, we assessed the binding of some recovered mAbs to antigens commonly used to assess autoimmune disease patients (Figure 6). Polyreactivity is commonly observed in mAbs and is a major feature of the natural B cell repertoire,^51^ thus it was not surprising to find that many recovered mAbs bound to autoantigens at low levels. We found that few tested mAbs showed strong binding to autoantigens, but we were surprised to find that the proportion of autoreactive antibodies decreased in later time points for the anti-Tac group compared with the CH65 controls. This suggested that Treg manipulation at later time points did not promote polyreactivity/autoreactivity.

The link between HIV-1-infection and autoimmune phenomena was observed soon after the start of the pandemic^52^ and the discordance of coincident HIV-1 infection and SLE^15^^,^^47^ led to numerous studies indicating a link between bnAb development and tolerance mechanisms.^5^^,^^13^^,^^53^^,^^54^ Development of bnAbs has been observed in 50% of HIV-1-infected individuals after years of infection but has not been reliably induced in uninfected individuals with HIV-1 Env vaccination.^55^^,^^56^ Those who make bnAbs have a lower frequency of Treg cells and a higher frequency of circulating Tfh cells,^15^ and Tregs suppress different types of immune responses to help maintain homeostasis and prevent autoimmunity and exaggerated immune responses. Tregs localized in the GC, Tfr cells, regulate GC responses and are crucial in controlling for autoimmune diseases.^57^ IL-2 inhibits Tfh cell differentiation through its effects on B cell lymphoma 6 protein^58^ and mTORC1,^59^ differentiation of the vaccine-primed CD4^+^ T cells might increase by blocking CD25 (IL-2RA). Given this evidence, it was logical to test whether Treg manipulation could enable enhanced B cell somatic hypermutation and repertoire diversification. As shown, anti-CD25 administration transiently decreased lymph node Treg and Tfr cells, mimicking the GC milieu thought to promote bnAb development. Unfortunately, anti-CD25 immune manipulation did not result in bnAb development but did have off target effects, mainly the development of anti-drug antibodies (Figure 7). Whether this was due only to the administration of anti-CD25 mAbs or to the combination of treatment and vaccination is yet to be determined. The presence of anti-IVIG plasma IgM in 2 of the 6 RMs later treated with anti-CD25 introduces complexity in interpreting whether the development of anti-drug antibodies resulted from a break in tolerance. However, we observed that the infusion of anti-CD25 monoclonal antibodies was associated with immunological changes indicative of tolerance disruption, including detectable anti-drug antibody responses. Despite the apparent stability in the frequencies of GC immune cell subsets (Figure 3), the cytokine or cellular milieu within lymph nodes might be disrupted, leading to a larger number of small clonal lineages and singletons (Figure 4), and a reduction in clonal expansions (Figure 5). These changes were observed predominantly after the first mAb infusion. Furthermore, the anti-IVIG IgM (rheumatoid factor) responses were boosted following repeated infusions, resembling patterns seen in autoimmune patients, which support the notion of a treatment-associated break in tolerance. However, this tolerance break appeared to be transient; the development of anti-drug antibodies ultimately blocked the therapeutic antibodies, limiting their capacity to manipulate Tregs and thereby diminishing their intended immunomodulatory effects, which may have contributed to the lack of desired outcomes (i.e., autoantibody production and bnAb development). Therefore, while these baseline responses raise the possibility of pre-existing reactivity, the subsequent development and boosting of anti-drug antibodies following anti-CD25 treatment suggest that tolerance was further disrupted by the treatment itself. Although we cannot formally prove causality between anti-antibody formation and loss of treatment efficacy, the temporal association between the emergence of anti-drug antibodies and the diminished immunomodulatory and vaccine effects suggests that anti-antibody responses may have compromised the activity of the infused mAbs.

Regardless, the observation may have important implications for the use of immune-modulating therapies around the time of vaccination, particularly in patients such as transplant recipients or those with autoimmune diseases who require both immune suppression and protection against infection. The rapid development of anti-drug antibodies in our study underscores the need to carefully consider the timing of antibody-based interventions. While anti-CD25 treatment transiently altered the GC response and suggested a break in tolerance, these effects were quickly counteracted by immune responses against the therapeutic antibodies themselves, limiting their sustained impact on Treg populations. These immune dynamics, although presenting challenges, offer valuable insights for future therapeutic strategies. In particular, they highlight the importance of optimizing the timing of drug administration relative to vaccination. Since bnAb development may involve transient autoreactivity and extended maturation periods, delaying immune-modulating antibody infusions until after key stages of vaccine-induced immune priming may reduce the development of anti-antibody responses and improve outcomes. Although our study design did not test varied infusion schedules, future studies may help define more favorable timing windows for balancing immune modulation and vaccine efficacy in clinical settings. Furthermore, this study provides another way to investigate off-target effects of vaccination in patients receiving immune modulating therapies and future studies may help define the best timing windows for the administration of vaccines and drugs. Continued exploration in this area will be essential to ensure both safety and effectiveness in populations requiring complex immune interventions. Such responses are not unexpected in nonhuman primate models and are consistent with reports of anti-drug antibodies in human patients treated with daclizumab in clinical settings. Anti-antibody responses to drugs used in multiple sclerosis has been seen, and humanized antibodies like daclizumab (as a fully humanized IgG1) can elicit anti-drug responses (5%–11%); these responses depend on the degree of humanization, and many other factors including dose, route, and target.^60^^,^^61^^,^^62^^,^^63^ Anti-drug effects also have been observed in the treatment of human diseases such as chronic hepatitis B infection where anti-drug antibodies developed to PegIFNa and reduced treatment effectiveness^64^; understanding how, why, and the timing of these responses may allow for optimization of treatment schedules.

### Limitations of the study

This study has several limitations. This was a pilot study, thus RM group sizes were small; this is appropriate given the expense of RM research and the need to carefully allocate a scarce resource, but it limits the ability to perform statistical analyses. It would be ideal to test with increased sample size in a future study to increase the statistical power. RMs are an outbred population, thus we observed significant heterogeneity of responses. There are also challenges of translating our findings from nonhuman primates to humans. One limitation of our study is the timing of lymph node collection, which was conducted at day 7 after each immunization. While this time point was selected based on prior data indicating that GC formation and early B cell selection events are already underway by this time,^65^ it is possible that key features of the primed GC response were not fully captured, such as peak clonal expansion or the onset of somatic hypermutation. Future studies incorporating multiple time points, including later sampling, would provide a more comprehensive view of the dynamics of B cell priming and maturation. Another limitation is that the mAbs we used in this study are not selective for antigen-specific Tregs and may affect other CD25^+^ populations, including activated T cells and Tfh cells, which are critical for optimal GC responses and the generation of bnAbs. This non-specific depletion approach introduces potential confounding effects. The mechanisms underlying bnAb induction are highly complex and likely involve multiple layers of regulation beyond what was captured in our model. Most autoreactive B cells are deleted during bone marrow development, potentially limiting the impact of peripheral tolerance modulation. In addition, further study of the impact of anti-CD25 treatment on antigen-specific T cell responses or GC immune cell function (i.e., cytokine profiles) in these RMs is not possible as all samples were consumed for the analyses presented. Future research should expand on our findings by employing more antigen-specific interventions and larger cohorts. To more rigorously test the hypothesis that modulation of immune tolerance could enhance bnAb development, future studies may benefit from the inclusion of epitopes known to be under tolerance control, such as the membrane-proximal external region (MPER) of gp41. The CH505 gp120 immunogen used here does not include MPER and thus cannot capture responses to this tolerance-sensitive site. Incorporating MPER-containing immunogens or immunogens that better mimic native trimeric Env may provide a more informative platform for evaluating the impact of Treg cell modulation on bnAb development. Nevertheless, we demonstrated that Treg perturbation during vaccination can alter some lymph node T cell populations, although the primary outcome was an anti-antibody response. It is possible that other Treg manipulation strategies may facilitate bnAb development, but it will be important to look for off target effects. Sex was not specifically analyzed in this study. This represents a limitation, and future investigations are warranted to explore their potential impact on the outcomes.

## Resource availability

### Lead contact

Further information and requests for resources and reagents should be directed to and will be fulfilled by Dr. M. Anthony Moody (moody007@mc.duke.edu).

### Materials availability

All unique/stable reagents generated in this study are available from the lead contact with a completed materials transfer agreement.

### Data and code availability

• All data reported in this article will be shared by the lead contact upon request.
• This article does not report original code.
• Any additional information required to reanalyze the data reported in this paper is available from the lead contact upon request.

## Acknowledgments

We thank Ahmad Yousef Abuahmad for expert technical assistance. We express our gratitude to Professor Persephone Borrow for her critical comments and suggestions. This work was supported by the National Institutes of Health, 10.13039/100000060National Institute of Allergy and Infectious Diseases, Division of AIDS grants Center For HIV/AIDS Vaccine Immunology-Immunogen Discovery (CHAVI-ID, UM1AI100645) and the Duke Consortia for HIV/AIDS Vaccine Development (CHAVD, UM1AI144371) both to B.F.H. Immunology support provided by the NIH Duke Center for AIDS Research (CFAR P30 AI064518) to G.D.T. The graphical abstract was created in BioRender. Gu, S. (2025) https://BioRender.com/o8r7663.

## Author contributions

B.F.H., H.-X.L., and M.A.M. conceived and designed the study. S.G., K.L., and M.A.M. wrote and edited the paper; all authors approved the final manuscript. S.G., K.L., T.A.V.H., L.L.S., R.P., and X.S. performed assays on plasma/serum and mAbs. K.L., T.C.G., T.A.V.H., and M.A.M. performed flow cytometry sorts and/or isolated antibodies. S.G., K.L., T.A.V.H., T.C.G., H.B.-V., X.S., R.L.S., G.D.T., D.C.M., H.-X.L., B.F.H., and M.A.M. analyzed data.

## Declaration of interests

The authors declare no competing interests.

## STAR★Methods

### Key resources table

**Table:** 

REAGENT or RESOURCE	SOURCE	IDENTIFIER
**Antibodies**		

basiliximab	Novartis	NDC 0078-0331-84
rhesusized daclizumab	Produced in house	N/A
influenza antibody CH65	Produced in house	N/A
BD Pharmingen™ PerCP-Cy™5.5 Mouse Anti-Human CD3 (clone SP-34-2)	BD Biosciences	Cat#: 552852; RRID: AB_394493
BD Pharmingen™ PE-Cy™7 Mouse Anti-Human CD16 (clone 3G8)	BD Biosciences	Cat#: 557744; RRID: AB_396850
BD™ FITC Mouse Anti-Human CD20 (clone L27)	BD Biosciences	Cat#: 347673; RRID: AB_400338
BD Pharmingen™ PE-Cy™5 Mouse Anti-Human IgM (clone G20-127)	BD Biosciences	Cat#: 551079; RRID: AB_394036
BD Horizon™ BV421 Mouse Anti-Human CD279 (PD-1) (clone EH12.1)	BD Biosciences	Cat#: 562516; RRID: AB_11153482
BD Pharmingen™ Alexa Fluor® 700 Mouse Anti-Human CD3 (clone SP34-2)	BD Biosciences	Cat#: 557917; RRID: AB_396938
Brilliant Violet 570™ anti-human CD8a Antibody (clone RPA-T8)	Biolegend	Cat#: 301037; RRID: AB_10933259
BD Horizon™ BV605 Rat Anti-Human CCR7 (CD197) (clone 3D12)	BD Biosciences	Cat#: 563711; RRID: AB_2738385
CD45RA Monoclonal Antibody (MEM-56), Qdot™ 655	Invitrogen	Cat#: Q10069; RRID: AB_2556451
Brilliant Violet 570™ anti-human CD14 Antibody (clone M5E2)	Biolegend	Cat#: 301832; RRID: AB_2563629
APC/Cyanine7 anti-human CD27 Antibody (clone O323)	Biolegend	Cat#: 302816; RRID: AB_571977
BD Horizon™ BV711 Mouse Anti-Human CD25 (clone 2A3)	BD Biosciences	Cat#: 563159; RRID: AB_2738037
PerCP/Cyanine5.5 anti-human/mouse/rat CD278 (ICOS) Antibody (clone C398.4A)	Biolegend	Cat#: 313518; RRID: AB_10641280
PE/Cyanine5 anti-human CD95 (Fas) Antibody (clone DX2)	Biolegend	Cat#: 305610; RRID: AB_314548
BD Pharmingen™ APC-Cy™7 Mouse Anti-Human CD154 (clone TRAP1)	BD Biosciences	Cat#: 563588; RRID: AB_2738296
PE anti-human CD150 (SLAM) Antibody (clone A12)	Biolegend	Cat#: 306308; RRID: AB_2187947
CD185 (CXCR5) Monoclonal Antibody (MU5UBEE), PE-Cyanine7	eBioscience	Cat#: 25-9185-42; RRID: AB_2573540
CD28-ECD (clone CD28.2)	Beckman Coulter	Cat#: 6607111; RRID: AB_1575955
Goat Anti-Human IgD-PE	Southern Biotech	Cat#: 2030-09; RRID: AB_2795630
CD8 Monoclonal Antibody (3B5), PE-Texas Red	Invitrogen	Cat#: MHCD0817; RRID: AB_10372359
CD4 Monoclonal Antibody (S3.5), PE-Cyanine5.5	Invitrogen	Cat#: MHCD0418; RRID: AB_10376013
FOXP3 Monoclonal Antibody (PCH101), APC	eBioscience	Cat#: 17-4776-42; RRID: AB_1603280
intravenous immune globulin	GAMMAGARD S/D	NDC 0944-2656-03
Monkey IgG gamma Antibody Peroxidase Conjugated	Rockland	Cat#: 617-103-012; RRID: AB_218715
Goat anti Monkey IgM (Fc specific), conjugated with Horseradish peroxidase	Exalpha	Cat#: GAMon/IgM(Fc)/PO

**Bacterial and virus strains**		

Pseudotyped virus strains including CH505s, CH505 w4.3, 57128.vrc15, Q168.a2, Q842.d12, MW965.26, 6644.v2.c33, SF162.LS, and SVA-MLV	Produced in house	N/A

**Biological samples**		

Rhesus macaque lymph nodes	Accessing Unit and Biorepository, Duke University	N/A
Rhesus macaque peripheral blood	Accessing Unit and Biorepository, Duke University	N/A
Rhesus macaque serum and plasma	Accessing Unit and Biorepository, Duke University	N/A

**Chemicals, peptides, and recombinant proteins**		

Envs gp120 CH505 transmitted/founder	Produced in house	N/A
natural gp120 CH505 variants (wk53, 78, and 100)	Produced in house	N/A
adjuvant GLA-SE	IDRI, now AAHI	N/A
CD25	Produced in house	N/A
HA A/Solomon Islands/03/2006	Produced in house	N/A
Peptide library (see Table S1)	Genscript	N/A

**Critical commercial assays**		

Effectene Transfection Reagent	QIAGEN	Cat#: 301425
HotStarTaq DNA Polymerase	QIAGEN	Cat#: 203203
KOD Hot Start DNA Polymerase	Novagen	Cat#: 80511-386

**Experimental models: Cell lines**		

Human: 293T cell line	ATCC	CRL-3216
TZM-bl cell line	NIH AIDS Research and Reference Reagent Program	Cat#: 8129

**Experimental models: Organisms/strains**		

Chinese Origin Rhesus macaques	BIOQUAL, Inc.	N/A

**Oligonucleotides**		

PCR primers (see Table S2)		

**Software and algorithms**		

FlowJo V10.0.7 software	TreeStar	https://www.flowjo.com
GraphPad Prism 7 software	GraphPad	https://www.graphpad.com
SPSS Statistics 20.0	IBM	https://www.ibm.com
Biorender	Biorender	https://biorender.com
Cloanalyst	Boston University Microbiology Laboratory of Computational Immunology	http://www.bu.edu/computationalimmunology/research/software/

**Other**		

Foxp3/Transcription Factor Fixation/Permeabilization Concentrate and Diluent	eBioscience	Cat#: 00-5521-00
Live/Dead Fixable Aqua Dead Cell Stain	Invitrogen	Cat#: L34957
Streptavidin, Alexa Fluor™ 647 Conjugate	Invitrogen	Cat#: S21374
Brilliant Violet 421™ Streptavidin	Biolegend	Cat#: 405226
SureBlue™ TMB 1-Component Microwell Peroxidase Substrate	KBL	Cat#: 5120-0077

### Experimental model and study participant details

#### Immunization of RMs

All RMs were housed at indoors at BIOQUAL, Inc., Rockville, MD, and were maintained in accordance with the Association for Assessment and Accreditation of Laboratory Animals with the approval of the IACUC. Research was conducted in compliance with the Animal Welfare Act and other federal statutes and regulations relating to animals and experiments involving animals and adheres to principles stated in the Guide for the Care and Use of Laboratory Animals, NRC Publication, 2011 edition. All animal experiments performed in this study were approved by the Duke and Bioqual IACUCs.

The vaccine regimen for this study is shown in Figure 1. Nine male rhesus macaques (RM) were randomly divided into three groups of 3 RMs per group which were vaccinated with sequential Envs CH505 transmitted/founder (TF), and natural CH505 variants (wk53, 78, and 100; 100μg IM) were given at days 0, 56, 112, and 168 respectively. All immunizations were given in divided dose in the quadriceps bilaterally; the adjuvant GLA-SE (IDRI, now AAHI) was coadministered for each immunization. RMs in group 1 were treated with basiliximab at 1 mg total dose five days after each immunization; those in group 2 were treated with 1 mg total dose of a rhesusized version of daclizumab (anti-Tac) on the same schedule; group 3 was treated with 1 mg of a rhesusized version of influenza antibody CH65 on the same schedule to serve as a negative control. Both CH65 and daclizumab used in this study were rhesusized by grafting the human variable regions onto rhesus IgG1 constant regions to minimize immunogenicity in RMs. Peripheral blood was obtained after each immunization and 5, 7, 12, 19, and 26 days; we also obtained blood at days −9 and 0. Lymph nodes from each RM were obtained by excisional biopsy: an axillary node was collected at day −9, inguinal nodes were collected one week after the first and second immunizations (days 7 and 63), and a final axillary node was collected one week after the third immunization (day 119). Excisional biopsies were obtained by alternating sides between procedures. Peripheral blood was processed for serum (coagulated blood) or plasma and PBMCs (days −16, days of mAb infusion and lymph node excision, and subsequently once per week for three consecutive weeks after each lymph node collection); all samples were aliquoted and maintained at −80°C (serum/plasma) or in the liquid nitrogen vapor phase (cells) for further analysis.

### Method details

#### Immune cell phenotypic analysis and HIV Env-specific B cell single-cell sorting by flow cytometry

Single-cell suspension from lymph node and peripheral blood mononuclear cells (PBMCs) were isolated and cryopreserved in liquid nitrogen using standard techniques until thawed for further analysis. Thawed lymph node cells or PBMCs were surface stained with directly fluorescent-labeled antibodies to CD3 PerCP-Cy5.5 (SP34-2), CD16 PE-Cy7 (3G8), CD20 FITC (L27), IgM PE-Cy5 (G20-127), PD 1 BV421 (EH12.1), CD3 AF700 (SP34-2), CCR7 BV605 (3D12), CD25 BV711 (2A3), CD154 APC-Cy7 (TRAP1), (all BD Biosciences); CD8 BV570 (RPA-T8), CD14 BV570 (M5E2), CD27 APC-Cy7 (O323), ICOS PerCP-Cy5.5 (C398.4A), CD95 PE-Cy5 (DX2), CD150 PE (A12) (all Biolegend); CXCR5 PE-Cy7 (MU5UBEE, eBioscience); CD28 ECD (CD28.2, Beckman Coulter); IgD PE (Southern Biotech); and CD45RA QdotTM 655 (MEM-56), CD8 PE Texas Red (3B5), CD4 PE-Cy5.5 (S3.5) (Invitrogen) at 4°C for 30 minutes; then washed and stained with Live/Dead Fixable Aqua Dead Cell Stain (Life Technologies) at room temperature for 10 minutes. Cells were then fixed and permeabilized at room temperature for 15 minutes using the FoxP3 Fix/Perm Kit (eBioscience) and stained in permeabilization buffer with a directly labeled antibody to Foxp3 APC (PCH101; eBioscience) at room temperature for 45 minutes, prior to two washes with permeabilization buffer. Flow cytometry data were acquired with a BD LSR II flow cytometer (BD Bioscience). All flow cytometric analyses were performed using FlowJo V10.0.7 software (Treestar).

For single cell isolation, cells were stained with a panel of fluorochrome-antibody conjugates and reagents to identify antigen-specific memory B cells. The panel consisted of Aqua Vital dye; CD3 PerCP-Cy5.5 (SP34-2), CD16 PE-Cy7 (3G8), CD20 FITC (L27) (all BD Biosciences); CD14 BV570 (M5E2), CD27 APC-Cy7 (O323) (both Biolegend); and IgD PE (Southern Biotech). Preformed conjugates for antigen-specific B cell sorting were made as described,^40^ using biotinylated gp120 protein coupled to streptavidin conjugates AlexaFluor 647 (Life Technologies) or BV421 (Biolegend). For better tracking the evolution of CH505 as confirmed previously,^33^ Sorting antigen-specific probes were double fluorescent gp120_CH505TF/d7_ for pre (day -9) and post 1st (day 7), or gp120_CH505TF Δ371_ for post 2nd (day 63) and post 3rd. Memory B cells were defined as CD3^−^CD14^−^CD16^−^CD20^+^IgD^−^; antigen-specific memory B cells positive for probes in both colors or gp120_CH505_ only were sorted as single cells into 96-well plates containing 20 μL/well reverse transcriptase (RT) buffer (Invitrogen) as described. Sorted plates were frozen immediately and maintained at −80°C before RT/PCR. Memory B cells that bind to gp120_CH505TF_ but not gp120_CH505TF Δ371_ were defined as CD4 binding site-specific memory B cells (differential binder).

#### PCR amplification of immunoglobulin V_H_ and V_L_ genes

The V_H_ and V_L_ genes of sorted single memory B cells were amplified as described. A 65°C/5-minute pre-RT incubation was performed by adding 0.1 μM of a mixture of immune globulin (Ig) constant region primers (including IgA, IgM, IgD, IgG, and IgE) to each well. Samples were chilled on ice and RT buffer was added following a 50°C/45min, 55°C/15min RT/PCR reaction. First round PCR (PCRa) of heavy (H), kappa (κ), and lambda (λ) chains was then performed using the cDNA product of RT/PCR. Each 50-μL reaction contained 5 μL cDNA, 0.6 μL HotStar Taq (Qiagen), 5 μL PCR Buffer (Qiagen), 10 μL Q Buffer (Qiagen), 0.4 μL 25 mM dNTPs (Qiagen), 25 mM MgCl2 (1 μL for IgH, 2 μL for Igκ and 3 μL for Igλ), and 0.125 μM IgH, Igκ, or Igλ variable region primer mixtures (Table S2). PCRa reaction conditions were as follows: 95°C/5min, (94°C/30s, 62°C [for IgH] or 64°C [for Igκ/λ]/45s, 72°C/90s) for 35 cycles, 72°C/7min, then hold at 10°C. PCRa products were used as templates for nested PCR (PCRb). Each 50 μL reaction contained 3 μL PCRa product, 0.6 μL HotStar Taq (Qiagen), 5 μL PCR Buffer, 10 μL Q Buffer, 0.4 μL 25 mM dNTPs, 25 mM MgCl2 (3 μL for IgH, 2 μl for Igκ/λ) and 0.125 μM IgH, IgK, or Igλ internal primers (Table S2). PCRb reaction conditions were the same as for PCRa above. PCRb products were then analyzed by 1.2% SYBER Safe E-Gels (Invitrogen). Wells with bands indicating a PCR product were purified and sequenced. Sequencing results were analyzed by Cloanalyst software (Boston University Microbiology Laboratory of Computational Immunology. http://www.bu.edu/computationalimmunology/research/software/. Accessed November 2, 2016.) to infer VDJ arrangements. Analysis of RM genes and clonal lineages was performed as described.^66^^,^^67^ Clonal lineage membership was determined by Cloanalyst, which takes into account V and J gene usage, CDR3 length, and overall sequence similarity. Cloanalyst reconstructs lineage relationships based on maximum posterior probability trees and groups sequences accordingly.

#### Recombinant mAb expression

For high-throughput characterization of isolated mAbs, transient transfection was performed as previously described. To produce the transfection construct, 2 μL VH/VL PCRb product, 1 μL KOD polymerase (Novagen), 5 μL KOD buffer, 5 μL dNTPs, 3 μL 25 mM MgSO_4_, 1 μL CMV-262, 1 μL 1822BGH (IgH) or BGH-1235 (Igκ/λ) (Table S2), 2 μL rhesus CMV_P DNA fragments, 2 μL reversed DNA primers for either IgH or Igκ/λ were brought to 50 μL with H_2_O. PCR conditions were as follows: 95°C/2 minutes, (95°C/20 seconds, 62°C/12 seconds, 70°C/60 seconds) for 30 cycles, then hold at 4°C. PCR products were purified by MinElute 96UF plates and analyzed by QIAxcel (Qiagen) before transient transfection. Transient transfection was performed using 293T cells cultured in 6-well plates. Approximately 1 μg of heavy and light chain recombinant linear fragments was cotransfected by Effectene (Qiagen) when cells cultured in 10% FBS supplemented DMEM were 80%–90% confluent. Cell medium was changed to 2% FBS/DMEM before adding the transfection mixture. After 3 days of incubation at 37°C/8% CO2, supernatants were harvested for analysis. The 293T cell line used in this study was obtained from ATCC. While ATCC perform routine authentication and quality control, including mycoplasma testing, we did not independently verify the identity of the cell lines or test for mycoplasma contamination during the course of this study.

#### ELISA assays of RM plasma and antibodies

Plasma samples were heat inactivated at 56°C for 5 minutes before ELISA assay. Samples of multiple time points (days 0, 5, 7, 12, 19, 26, 61, 63, 69, 75, 82, 117, 119 and 124) were tested HIV-1 Env binding against antigens included HIV-1 antigen gp120_CH505TF_ (including the WT and the CD4bs mutants Δ371) and RSC3 (a recombinant gp120-derived protein with enhanced stability and accurate maintenance of the correct structure of the CD4bs, facilitating targeted recognition of the CD4bs while rendering the rest of gp120 unfavorable for recognition by altering or masking immunogenic epitopes; including the WT strain and the CD4bs mutants Δ371/P363N); binding against infused mAbs (Basiliximab, anti-Tac and CH65) were tested in the same way. Interaction against autoantigens (including double-stranded deoxyribonucleic acid (dsDNA), centromere B, histones, histidine-tRNA ligase (Jo1), Sjögren’s syndrome-related antigen A (SSA) and B (SSB), topoisomerase I (Scl-70), Smith antigen (Sm), ribonucleoprotein (RNP)) were tested on day -9, 7, 63 and 119 samples of each RM. Antigens were coated at 4°C on 384-well Costar high-binding ELISA plates at 30 ng per well. After overnight incubation, ELISA plates were blocked by Superblock (40 g whey protein, 150 mL goat serum, 5.0 ml Tween-20, 0.5 g NaN3, and 40 mL 25× PBS brought to 1 L with deionized water) for 1 hour at room temperature. Plasma was diluted 3-fold for 12 dilutions in Superblock buffer and added at 10 μL per well. After incubated at room temperature for 1 h, plates were washed twice with PBS containing 0.1% Tween-20. Horseradish peroxidase–conjugated goat anti-monkey secondary antibodies (Rockland, catalog 617-103-012) were diluted 1:10,000 in Superblock and added at 10 μL per well for one hour incubation. Plates were washed 4 times before 20 μL of SureBlue Reserve TMB-1 Solution (KPL) was added. After a 15 min incubation, the reaction was stopped with 20 μL 1% HCl. Plates were read by a SpectraMax Plus 384 plate reader (Molecular Devices) at 450 nm. For plasma binding assays, area under the curve (AUC) was calculated using the trapezoidal method. For testing of expressed mAbs, positivity thresholds for binding to Env proteins were set at 0.25 Absorbent unit (AU), > 4-fold over background; thresholds for autoantigens were >0.75 AU for strongly autoreactive, 0.5-0.75 AU for mildly autoreactive, and 0.25-0.5 AU for weakly autoreactive.

Rheumatoid Factor-IgM antibodies were also measured with an in-house ELISA, using intravenous immune globulin (IVIG, GAMMAGARD) as the antigen, which was diluted to 2 μg/mL in Biocarbonate buffer and incubated overnight at 4°C. Plates were blocked for 1 hour at room temperature by Superblock. Serum samples started at a 1:30 dilution then use a 2-fold dilution in Superblock and incubated for 1 hour at 37 °C. Antibody binding was detected with Goat anti Monkey IgM (Fc specific) conjugated with Horseradish peroxidase (GAMon/IgM(Fc)/PO, Exalpha).

Competitive inhibition ELISAs were performed as previously described using the same plasma samples at 1:50 dilution and using gp120CH505 and gp120_63521_ as the target antigen. HIV-1 mAbs CH106 and sCD4 were tested.

For VH and VL transient transfection supernatants, screening was performed as described using the following HIV-1 antigens: gp120_CH505TF_ (WT, Δ371); RSC3 (WT, Δ371/P363N); gp120YU2 (WT, D368R); sequential gp120_CH505_ (week53, week 78 and week 100) and CONS V3. The concentration of mAb in each supernatant was quantified using a capture ELISA method as previously described; known concentrations of mAb 2F5 were used to generate the standard curve. Supernatants were undiluted for ELISA binding screening.

#### Neutralization assay of antibodies in sera

Serum samples from days 0, 124, 187, and 329 were heat inactivated at 56°C for 5 minutes and tested for neutralization activity starting at 1:10 dilution (3-fold dilution). Pseudotyped virus strains including CH505s, CH505 w4.3, 57128.vrc15, Q168.a2, Q842.d12, MW965.26, 6644.v2.c33, SF162.LS, as well as SVA-MLV as a control, were tested using TZM-bl cells. Inhibitory dilution 50% (ID50) as reciprocal dilution for serum neutralization activity was calculated using logistic regression. For Ab precursor analysis, Env-pseudotyped virus strains including CH505TF.G458Y.N279K, CH505TF.G458Y.N279K.N280D, CH505TF.gly4, and CH505TF.gly4.S365P were tested on day 124 and day 187 sera. The TZM-bl cells used in this study were obtained from NIH AIDS Research and Reference Reagent Program. While they perform routine authentication and quality control, including mycoplasma testing, we did not independently verify the identity of the cell lines or test for mycoplasma contamination during the course of this study.

#### Linear epitope mapping of plasma antibodies

Peptide microarray analysis was performed using a Tecan HS4000 Hybridization Workstation reported previously. Peptide library consisting of 15-mers overlapped by 12 aa were printed onto glass slides (JPT, ES-025), covering the full length of consensus gp120 Env from vaccine strains including A244, TH023, MN, C.1086, C.TV1, C.ZM651, and 59 CH505 strains including CH505.TF (). Plasma samples from day 0, day 12, day 69 and day 124 of each RM were tested at 1:50 dilution. Goat anti human IgG conjugated with Alexa Fluor 647 was used at 1:500 dilution as secondary antibody. XDR accurate mode was used during the scan. Each samples are tested against all peptides in triplicates. Peptide binding signals are qualified as Log2-transformed chemifluorescence intensity of the post vaccination sample subtracted by Log2-transformed chemifluorescence intensity of the matched baseline sample.

### Quantification and statistical analysis

Kruskal-Wallis H tests were used when more than two groups were compared, performing a post hoc test (Dunn’s test) that applies correction for multiple comparisons. SPSS Statistics 20.0 (Chicago, IL) and GraphPad Prism 7 software were used for statistical analysis. A *p* value of less than 0.05 was considered significant.

## Supplemental information

Document S1. Figures S1–S7

Table S1. Peptide sequence, related to STAR Methods

Table S2. Primers for PCR, related to STAR Methods
