# Supplementary material for: Transient regulatory T cell manipulation is limited by anti-antibody responses in HIV-1 envelope immunized rhesus macaques
Source: iScience. 2025 Jul 23;28(8):113191. doi: 10.1016/j.isci.2025.113191 (PMC12355595; doi:10.1016/j.isci.2025.113191)
Supplement: Document S1. Figures S1–S7 [file mmc1.pdf]

iScience, Volume *28*

Supplemental information

Transient regulatory T cell manipulation is limited by anti-antibody responses in HIV-1 envelope immunized rhesus macaques

Shuqin Gu, Kan Luo, Tarra A. Von Holle, Thaddeus C. Gurley, Hilary Bouton-Verville, Laura L. Sutherland, Robert Parks, Xiaoying Shen, Rachel L. Spreng, Georgia D. Tomaras, David C. Montefiori, Hua-Xin Liao, Barton F. Haynes, and M. Anthony Moody

Figure S1

A

Cohort	Animal ID	Timing of serum/plasma samples (days)																									
		-16	-9	0	5	7	12	19	26	56	61	63	69	75	82	112	117	119	124	130	137	168	173	175	180	187	196	329
basiliximab (1mg)	6117	-16	-9	/	5	7	12	19	26	/	61	63	69	75	82	/	117	119	124	130	137	/	173	175	180	187	196	329
	6160	-16	-9	/	5	7	12	19	26	/	61	63	69	75	82	/	117	119	124	130	137	/	173	175	180	187	196	329
	6204	-16	-9	/	5	7	12	19	26	/	61	63	69	75	82	/	117	119	124	130	137	/	173	175	180	187	196	329
anti-Tac (1mg)	6105	-16	-9	/	5	7	12	19	26	/	61	63	69	75	82	/	117	119	124	130	137	/	173	175	180	187	196	329
	6120	-16	-9	/	5	7	12	19	26	/	61	63	69	75	82	/	117	119	124	130	137	/	173	175	180	187	196	329
	6125	-16	-9	/	5	7	12	19	26	/	61	63	69	75	82	/	117	119	124	130	137	/	173	175	180	187	196	329
CH65 control (1mg)	6107	-16	-9	/	5	7	12	19	26	/	61	63	69	75	82	/	117	119	124	130	137	/	173	175	180	187	196	329
	6200	-16	-9	/	5	7	12	19	26	/	61	63	69	75	82	/	117	119	124	130	137	/	173	175	180	187	196	329
	6203	-16	-9	/	5	7	12	19	26	/	61	63	69	75	82	/	117	119	124	130	137	/	173	175	180	187	196	329
	Event		Lymph nodes	Immunization	mAb infusion	Lymph nodes				Immunization	mAb infusion	Lymph nodes				Immunization	mAb infusion	Lymph nodes				Immunization	mAb infusion					

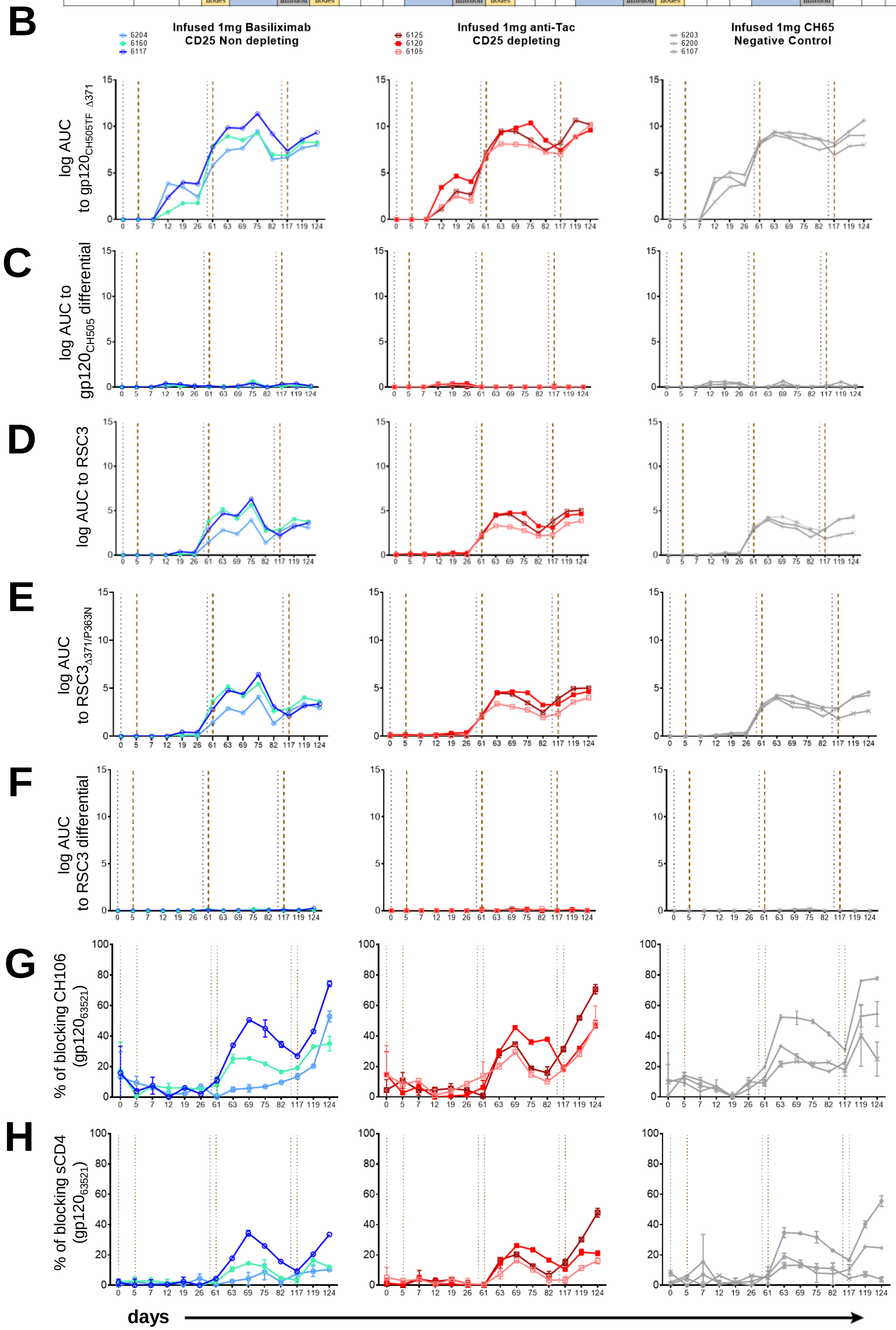

Figure S2

A

ID50 titer in TZM.bl cells

Virus	Tier	Clade	Group 1: Basiliximab											
			6160				6117				6204			
			pre	d124	d187	d329	pre	d124	d187	d329	pre	d124	d187	d329
CH0505.w4.3	1b	C	<20	209	141	2242	<20	1059	1019	3783	<20	194	127	1539
CH0505s	2	C	<20	<20	<20	<20	<20	<20	<20	<20	<20	<20	<20	<20
SVA-MLV		control	<20	<20	<20	<20	<20	<20	<20	<20	<20	<20	<20	<20
SF162.LS	1a	B	<20	<20	<20	<20	<20	768	414	208	<20	20	<20	<20
MW965.26	1a	C	<20	456	274	2231	<20	1011	1117	2173	<20	360	346	901
6644.v2.c33	1b	C	<20	26	<20	28	<20	34	<20	<20	<20	33	<20	<20
Q842.d12	2	A	<20	<20	<20	<20	<20	<20	<20	<20	<20	<20	<20	<20
57128.vrc15	2	D	<20	<20	<20	<20	<20	28	<20	<20	<20	<20	<20	<20
Q168.a2	2	A1D	<20	<20	<20	<20	<20	<20	<20	<20	<20	<20	<20	<20

Virus	Group 2: rhesusized anti-Tac											
	6105				6120				6125			
	pre	d124	d187	d329	pre	d124	d187	d329	pre	d124	d187	d329
CH0505.w4.3	<20	234	475	2656	<20	426	157	1922	<20	489	329	4474
CH0505s	<20	<20	<20	<20	<20	<20	<20	21	<20	<20	<20	<20
SVA-MLV	<20	<20	<20	<20	<20	<20	<20	<20	<20	<20	<20	<20
SF162.LS	<20	28	<20	<20	<20	61	<20	27	<20	173	40	49
MW965.26	<20	278	485	1231	<20	876	448	1328	<20	707	535	1721
6644.v2.c33	<20	33	<20	<20	<20	46	<20	29	<20	50	<20	42
Q842.d12	<20	<20	<20	<20	<20	<20	<20	<20	<20	<20	<20	<20
57128.vrc15	<20	<20	<20	<20	<20		<20	<20	<20		<20	<20
Q168.a2	<20	<20	<20	<20	<20	<20	<20	<20	<20	<20	<20	<20

Virus	Group 3: CH65											
	6107				6203				6200			
	pre	d124	d187	d329	pre	d124	d187	d329	pre	d124	d187	d329
CH0505.w4.3	<20	416	483	4541	<20	496	117	2187	<20	695	539	2634
CH0505s	<20	<20	<20	<20	<20	<20	<20	<20	<20	<20	<20	<20
SVA-MLV	<20	<20	<20	<20	<20	<20	<20	<20	<20	<20	<20	<20
SF162.LS	<20	<20	<20	<20	<20	<20	<20	<20	<20	83	25	57
MW965.26	<20	1873	2823	5156	<20	420	790	1337	<20	966	966	1574
6644.v2.c33	<20	42	<20	129	<20	22	<20	46	<20	52	22	43
Q842.d12	<20	<20	<20	<20	<20	<20	<20	<20	<20	<20	<20	<20
57128.vrc15	<20	<20	<20	<20	<20	<20	<20	<20	<20	<20	<20	<20
Q168.a2	<20	<20	<20	<20	<20	<20	<20	<20	<20	<20	<20	<20

B

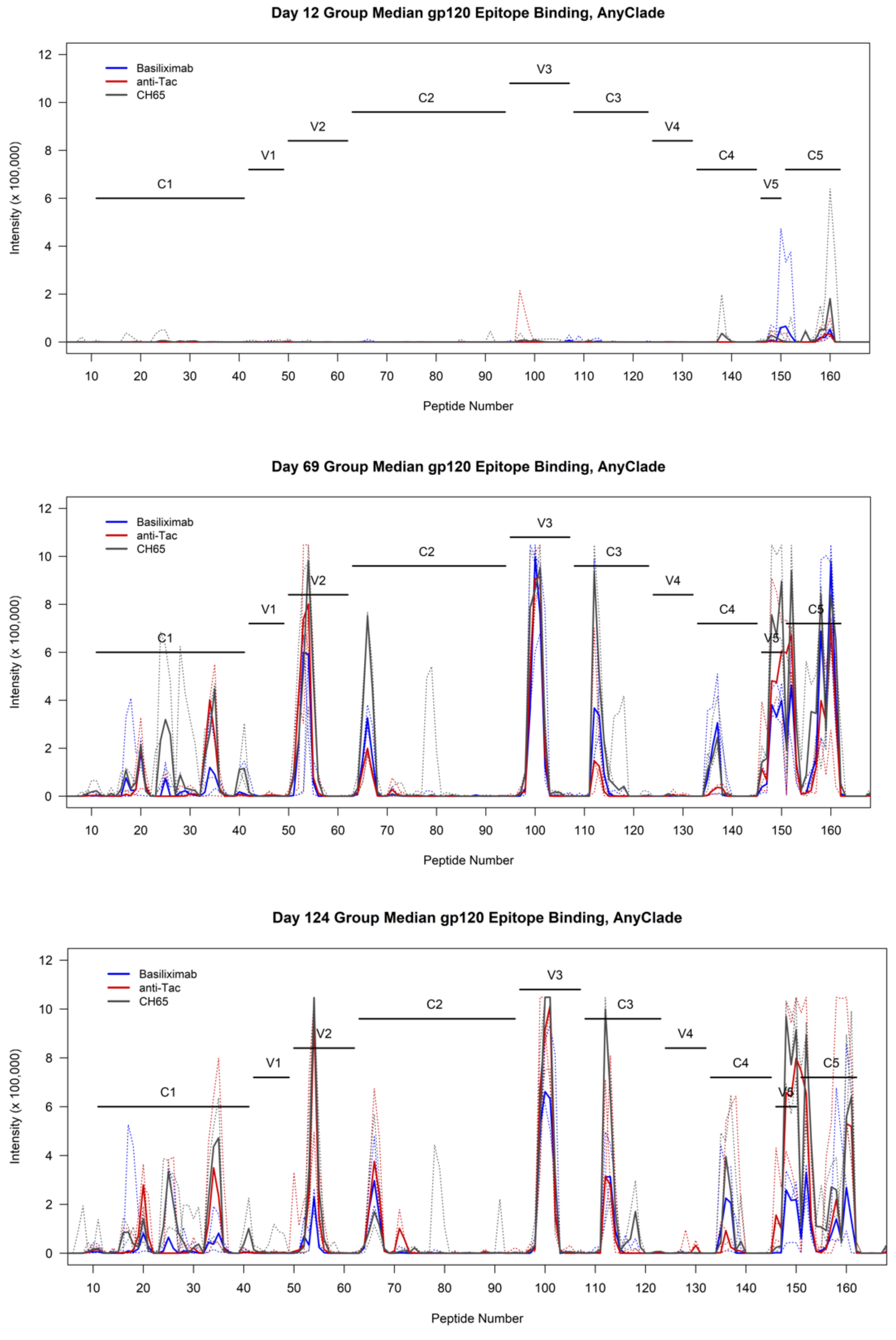

Figure S3

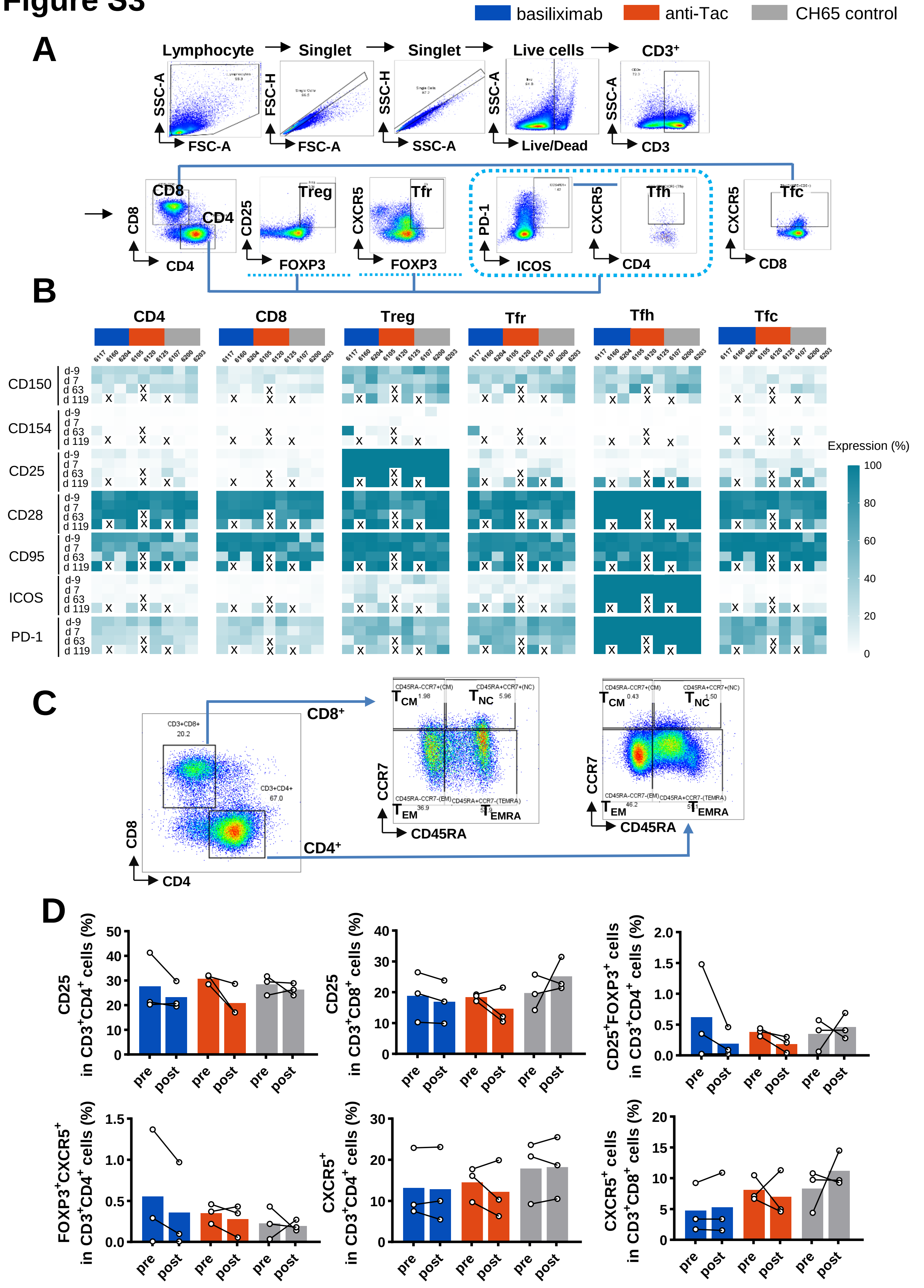
basiliximab
anti-Tac
CH65 control
A
Lymphocyte
Singlet
Singlet
Live cells
CD3+
SSC-A
FSC-A
FSC-H
FSC-A
SSC-H
SSC-A
SSC-A
Live/Dead
SSC-A
CD3
CD8
CD4
CD8
CD4
Treg
CD25
FOXP3
Tfr
CXCR5
FOXP3
PD-1
ICOS
Tfh
CXCR5
CD4
Tfc
CXCR5
CD8
B
CD4
CD8
Treg
Tfr
Tfh
Tfc
6117 6160 6204 6105 6120 6125 6107 6200 6203
CD150
CD154
CD25
CD28
CD95
ICOS
PD-1
d-9
d 7
d 63
d 119
Expression (%)
100
80
60
40
20
0
C
CD3+CD8+
20.2
CD3+CD4+
67.0
CD8+
CD4+
CD8
CD4
CD45RA-CCR7+(CM)
CD45RA+CCR7+(NC)
TCM
TNC
1.98
5.96
CD45RA-CCR7-(EM)
CD45RA+CCR7-(TEMRA)
TEM
TEMRA
36.9
CCR7
CD45RA
0.43
1.50
46.2
D
CD25 in CD3+CD4+ cells (%)
CD25 in CD3+CD8+ cells (%)
CD25+FOXP3+ cells in CD3+CD4+ cells (%)
FOXP3+CXCR5+ in CD3+CD4+ cells (%)
CXCR5+ in CD3+CD4+ cells (%)
CXCR5+ cells in CD3+CD8+ cells (%)
pre
post

Figure S4

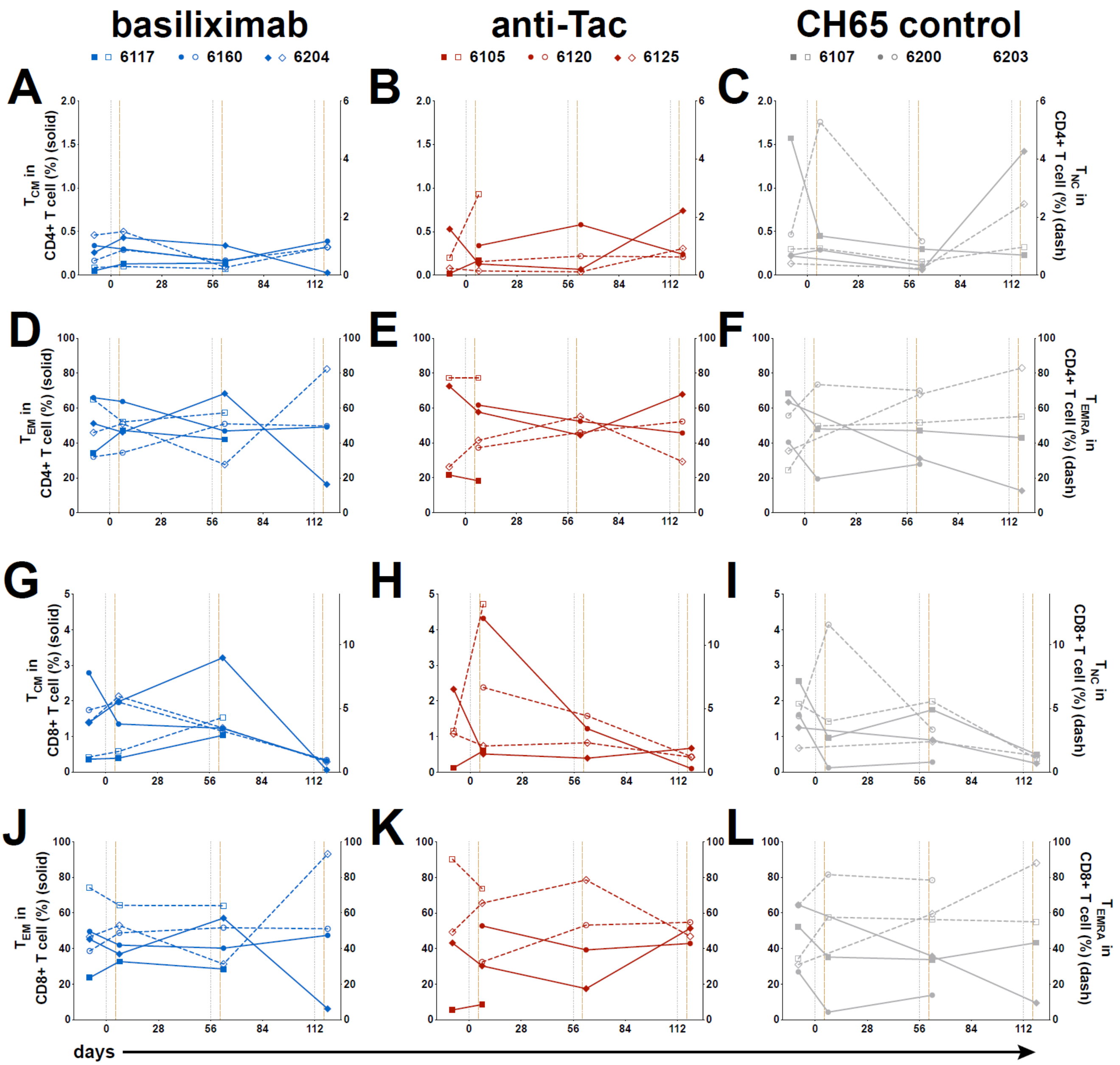
basiliximab
6117
6160
6204
anti-Tac
6105
6120
6125
CH65 control
6107
6200
6203
A
B
C
D
E
F
G
H
I
J
K
L
T_CM in CD4+ T cell (%) (solid)
T_NC in CD4+ T cell (%) (dash)
T_EM in CD4+ T cell (%) (solid)
T_EMRA in CD4+ T cell (%) (dash)
T_CM in CD8+ T cell (%) (solid)
T_NC in CD8+ T cell (%) (dash)
T_EM in CD8+ T cell (%) (solid)
T_EMRA in CD8+ T cell (%) (dash)
days

Figure S5

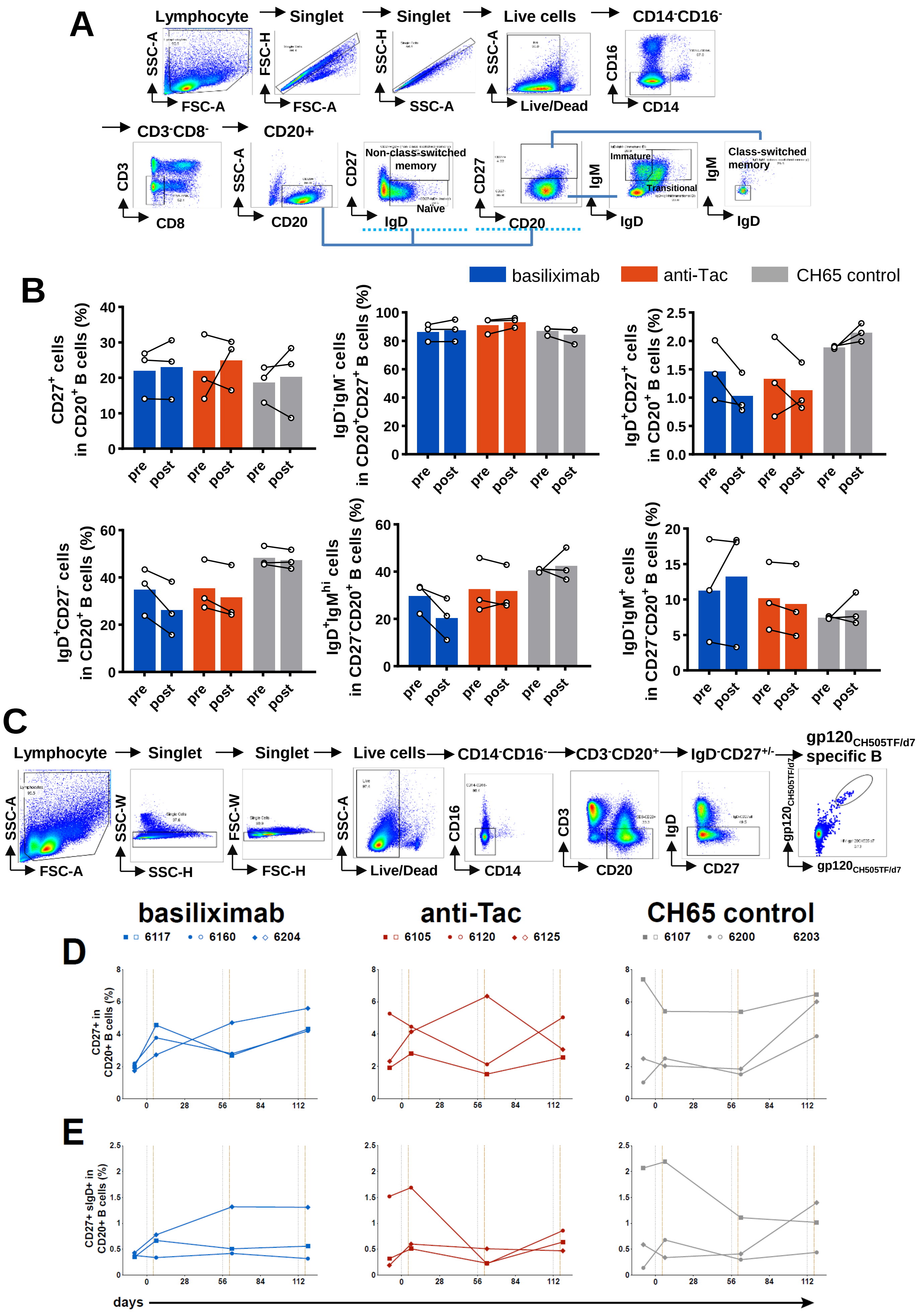
A
Lymphocyte
Singlet
Singlet
Live cells
CD14-CD16-
SSC-A
FSC-A
FSC-H
SSC-H
Live/Dead
CD16
CD14
CD3-CD8-
CD20+
CD3
CD8
CD20
CD27
IgD
Non-class-switched memory
Naive
IgM
Immature
Transitional
Class-switched memory
B
basiliximab
anti-Tac
CH65 control
CD27+ cells in CD20+ B cells (%)
IgD-IgM- cells in CD20+CD27+ B cells (%)
IgD+CD27+ cells in CD20+ B cells (%)
IgD+CD27- cells in CD20+ B cells (%)
IgD+IgMhi cells in CD27-CD20+ B cells (%)
IgD-IgM+ cells in CD27-CD20+ B cells (%)
pre
post
C
Lymphocyte
Singlet
Singlet
Live cells
CD14-CD16-
CD3-CD20+
IgD-CD27+/-
gp120CH505TF/d7 specific B
SSC-A
FSC-A
SSC-W
SSC-H
FSC-W
FSC-H
Live/Dead
CD16
CD14
CD3
CD20
IgD
CD27
gp120CH505TF/d7
basiliximab
6117
6160
6204
anti-Tac
6105
6120
6125
CH65 control
6107
6200
6203
D
CD27+ in CD20+ B cells (%)
E
CD27+ sIgD+ in CD20+ B cells (%)
days
0
28
56
84
112

Figure S6

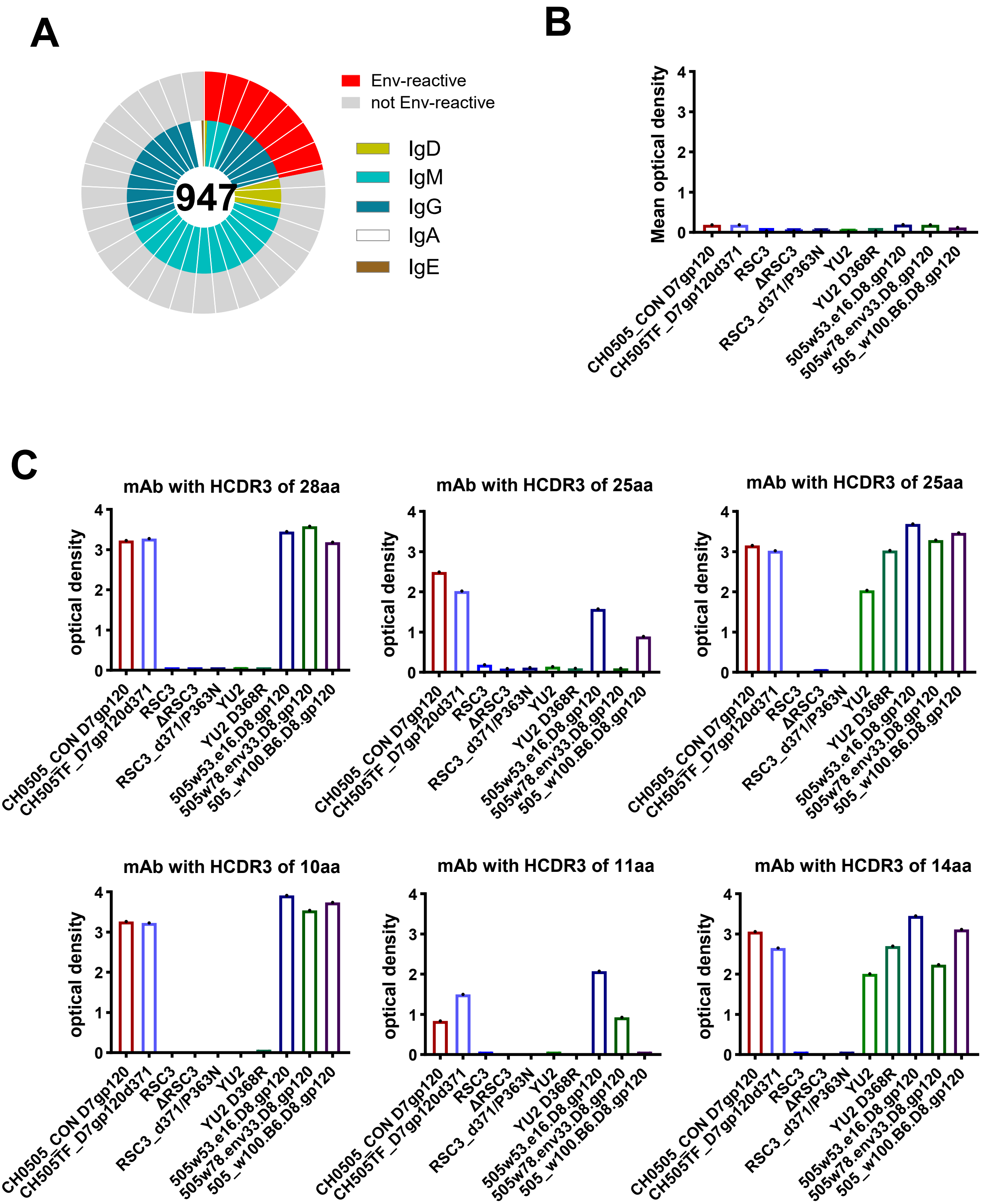

A
947
Env-reactive
not Env-reactive
IgD
IgM
IgG
IgA
IgE
B
Mean optical density
CH0505_CON D7gp120
CH505TF_D7gp120d371
RSC3
ΔRSC3
RSC3_d371/P363N
YU2
YU2 D368R
505w53.e16.D8.gp120
505w78.env33.D8.gp120
505_w100.B6.D8.gp120
C
mAb with HCDR3 of 28aa
mAb with HCDR3 of 25aa
mAb with HCDR3 of 25aa
mAb with HCDR3 of 10aa
mAb with HCDR3 of 11aa
mAb with HCDR3 of 14aa
optical density

Figure S7

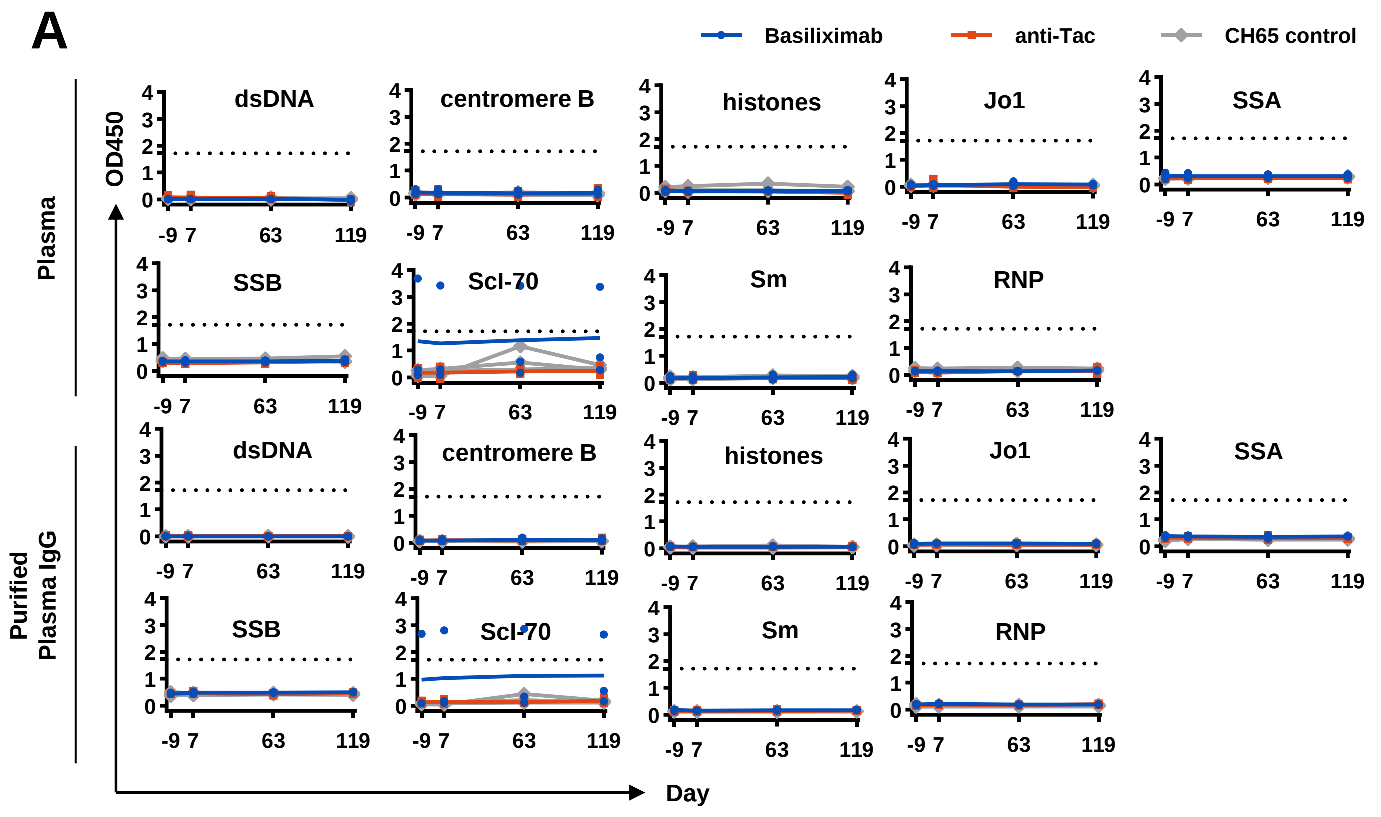

A
Basiliximab
anti-Tac
CH65 control
Plasma
Purified Plasma IgG
OD450
Day
dsDNA
centromere B
histones
Jo1
SSA
SSB
Scl-70
Sm
RNP
0
1
2
3
4
-9
7
63
119

Figure S1. Binding and blocking activity of antibodies in plasma, related to Figure 1. (**A**) Tabular version of the experimental timeline. Plasma from subjects of each group was collected from a series of time points (X-axis) and tested the binding against HIV-1 Envs $gp120_{CH505TF\ \Delta 371}$ (**B**), RSC3 (**D**), and $RSC3_{\Delta 371/P363N}$ (**E**). CD4bs differential binding activity on $gp120_{CH505TF}$ vs. $gp120_{CH505TF\ \Delta 371}$ (**C**) and RSC3 vs. $RSC3_{\Delta 371/P363N}$ (**F**). Log AUC on Y-axis indicated the strength of plasma binding activity. Blocking against CH106 to $gp120_{63521}$ was shown in **G** and blocking against sCD4 was shown in **H**. RMs in Basiliximab, anti-Tac, and CH65 control mAb-treated groups were shown respectively. AUC, area under the curve; CD4bs, CD4 binding site; Envs, envelops; sCD4, soluble CD4; RMs, rhesus macaques; RSC3, resurfaced stabilized core 3.

Figure S2. Neutralization and linear epitope mapping of plasma antibodies, related to Figure 2. (**A**) Plasma samples collected on pre-immunization, days 124, 187, and 329 were heat inactivated before being tested against a series of pseudotyped viruses. ID50 titer was exhibited. (**B**) Highest binding to peptide of any clade (including consensus Clade A, B, C, D, CRF01_AE, CRF02_AG, Group M; and from vaccine strains including A244, TH023, MN, C.1086, C.TV1, C.ZM651, and CH505TF) in gp120 in all CH505 sequential immunized groups. Thick lines are group median binding intensity values; thin lines are binding by individual animals, color-coded by a group. Intensity values are background subtracted. Peptide numbers are aligned to Con M sequences. Amino acid positions (based on HXB2 numbers) are each peptide number and the peptide sequences for Con M and CH505TF are given in Supplemental Table 1.

Figure S3. Surface molecules expression on germinal center cells and T/B cell characteristics in PBMC, related to Figure 3. (**A**). Gating strategy for regulatory T (Treg, $FoxP3^{+}$ $CD25^{+}$ $CD4^{+}$), follicular regulatory T (Tfr, $FoxP3^{+}$ $CXCR5^{+}$ $CD4^{+}$), follicular helper T (Tfh, $ICOS^{+}$ $PD\text{-}1^{+}$ $CXCR5^{+}$ $CD4^{+}$), and follicular cytotoxic T (Tfc, $CXCR5^{+}$ $CD8^{+}$) cells. (**B**). Heat map illustrating the expression of the indicated surface molecules. No detection (label X) at d119 for #6160, #6120, #6107, and at d63 for #6120 for all panels. Gating strategy of central memory T (T_{CM}, $CCR7^{+}$ $CD45RA^{-}$), Effector memory T (T_{EM}, $CCR7^{-}$ $CD45RA^{-}$), naïve T (T_{NC}, $CCR7^{+}$ $CD45RA^{+}$), and terminally differentiated effector memory T cells (T_{EMRA}, $CCR7^{-}$ $CD45RA^{+}$) were shown in **C**. CD25 expression on Treg cells and ICOS and PD-1 expression on Tfh cells are 100%. CD25 expression on $CD4^{+}$ and $CD8^{+}$ T cells, and percentage of Treg, Tfr, Tfh, and Tfc cells in PBMC pre and post the first infusion were shown (**D**). PBMC, peripheral blood mononuclear cell.

Figure S4. T-cell subsets kinetics, related to Figure 3. Percentage of central memory T (T_{CM}, $CCR7^{+}$ $CD45RA^{-}$), Effector memory T (T_{EM}, $CCR7^{-}$ $CD45RA^{-}$), naïve T (T_{NC}, $CCR7^{+}$ $CD45RA^{+}$), and terminally differentiated effector memory T cells (T_{EMRA}, $CCR7^{-}$ $CD45RA^{+}$). Gray dot lines indicated immunization time points, and brown lines represented the time point that RMs received mAb infusion. The solid lines illustrate T_{CM} or T_{EM} in $CD4^{+}$ or $CD8^{+}$ T cells, while the dash lines displays T_{NC} or T_{EMRA} in $CD4^{+}$ or $CD8^{+}$ T cells.

Figure S5. B-cell subsets kinetics, related to Figure 4. (**A**) Gating strategy of memory B ($CD27^{+}$ $CD20^{+}$), class-switched B (IgD^{-} IgM^{-} $CD27^{+}$ $CD20^{+}$), non-class-switched B (IgD^{+} $CD27^{+}$ $CD20^{+}$), naïve B (IgD^{+} $CD27^{-}$ $CD20^{+}$), transitional B (IgD^{+} IgM^{hi} $CD27^{-}$ $CD20^{+}$), and immature B (IgD^{-} IgM^{+} $CD27^{-}$ $CD20^{+}$) cells. (**B**) Frequencies of memory B, class-switched B, non-class-switched B, naïve B, transitional B, and immature B cells in PBMC pre and post the first infusion. (**C**) Sorting strategy for Env gp120-specific B cells. Env gp120-specific B cells was determined by dual staining with fluorochrome-conjugated $gp120_{CH505}$. Frequencies of $CD27^{+}$ $CD20^{+}$ B cells (**D**) and non-class-switched memory B cells in lymph nodes (**E**). PBMC, peripheral blood mononuclear cell.

Figure S6. Characteristics of mAbs, related to Figures 4 and 5. (**A**) Env-reactive mAbs isolated from basiliximab and anti-Tac groups are shown. The inner ring shows antibody isotype; the outer ring shows Env-reactivity. (B) Binding activity of mAbs with high degrees of mutation (Mutation frequency >20%, n=40). (C) Binding activity of mAbs with different HCDR3.

Figure S7. Polyreactivity of plasma antibodies and purified plasma IgG, related to Figures 3 and 6. Polyreactivity of plasma (upper panel) and purified plasma IgG (lower panel) were showed. Nine human autoantigens were tested against RMs samples on days -9, 7, 63, and 119 (pre-immunization and 7 days post each vaccination, X-axis). OD450 on Y-axis and the positive cutoff line was shown by the dash line. Individuals from Basiliximab, anti-Tac and control groups were shown in blue, red, and gray dots

respectively. The line indicates the average binding strength of each group. OD450, optical density at 450 nm; RMs, rhesus macaques.
